# Thermal Bioprinting Causes Ample Alterations of Expression of LUCAT1, IL6, CCL26, and NRN1L Genes and Massive Phosphorylation of Critical Oncogenic Drug Resistance Pathways in Breast Cancer Cells

**DOI:** 10.3389/fbioe.2020.00082

**Published:** 2020-02-21

**Authors:** Aleli Campbell, Jonathon E. Mohl, Denisse A. Gutierrez, Armando Varela-Ramirez, Thomas Boland

**Affiliations:** ^1^Metallurgical, Materials and Biomedical Engineering, University of Texas at El Paso, El Paso, TX, United States; ^2^Department of Mathematical Sciences and Border Biomedical Research Center, University of Texas at El Paso, El Paso, TX, United States; ^3^Department of Biological Sciences, Border Biomedical Research Center, University of Texas at El Paso, El Paso, TX, United States

**Keywords:** bioprinting, tumor model, molecular properties, drug discovery, kinase phosphorylation

## Abstract

Bioprinting technology merges engineering and biological fields and together, they possess a great translational potential, which can tremendously impact the future of regenerative medicine and drug discovery. However, the molecular effects elicited by thermal inkjet bioprinting in breast cancer cells remains elusive. Previous studies have suggested that bioprinting can be used to model tissues for drug discovery and pharmacology. We report viability, apoptosis, phosphorylation, and RNA sequence analysis of bioprinted MCF7 breast cancer cells at separate timepoints post-bioprinting. An Annexin A5-FITC apoptosis stain was used in combination with flow cytometry at 2 and 24 h post-bioprinting. Antibody arrays using a Human phospho-MAPK array kit was performed 24 h post-bioprinting. RNA sequence analysis was conducted in samples collected at 2, 7, and 24 h post-bioprinting. The post-bioprinting cell viability averages were 77 and 76% at 24 h and 48 h, with 31 and 64% apoptotic cells at 2 and 24 h after bioprinting. A total of 21 kinases were phosphorylated in the bioprinted cells and 9 were phosphorylated in the manually seeded controls. The RNA seq analysis in the bioprinted cells identified a total of 12,235 genes, of which 9.7% were significantly differentially expressed. Using a ±2-fold change as the cutoff, 266 upregulated and 206 downregulated genes were observed in the bioprinted cells, with the following 5 genes uniquely expressed NRN1L, LUCAT1, IL6, CCL26, and LOC401585. This suggests that thermal inkjet bioprinting is stimulating large scale gene alterations that could potentially be utilized for drug discovery. Moreover, bioprinting activates key pathways implicated in drug resistance, cell motility, proliferation, survival, and differentiation.

## Introduction

*In vitro* testing for drug discovery keeps making strides, especially with the advancement of genomics, proteomics, pharmacodynamics, bioinformatics, and automated High Throughput Screening (Andrade et al., 2016; Peng et al., 2017). Target-based drug design using appropriate cell assays, has not only transformed the identification of new targets, but it has also been supplemented with virtual testing aka “*in silico*” testing. With this approach, computer-based methods for drug simulations are utilized (Andrade et al., 2016; Hevener, 2018). *In silico* methods provide rapid and inexpensive techniques for quick lead test verification which proceed with *in vitro* cell testing. This method is a critical step in preclinical studies (Swinney and Anthony, 2011; Begley and Ellis, 2012; Peng et al., 2016). Previous studies have suggested that bioprinting can be used to model tissues for drug discovery and pharmacology (Peng et al., 2016, 2017). Peng et al., suggested that 3D bioprinting can help reduce the attrition rate in drug discovery by creating more realistic models. Through manipulation of pattern or anatomical models, it is possible to create permeable structures that ensure adequate delivery of nutrients and vascularization, which is primordial of *in vivo* environments. By bioprinting realistic models, we mean to generate tissue based on specific targeted characteristics such as lung, bone, cardiac, and even tumors.

While it is important to have a clear insight regarding cell viability and physiological changes of bioprinted (BP) cells, it is critical to understand the molecular changes within these cells in order to identify triggering mechanisms associated with cellular functions and behaviors. To our knowledge, this type of analysis has not been published before. Zhao et al., tested a 3D extrusion based bioprinted model of HeLa cells and found morphological differences, increased matrix metalloproteinase protein expression and higher cell proliferation when compared to the 2D standard cell culture. It is important to comprehend the gross anatomical structure as well as intra-cellular alterations to be able to model external stimuli, either of biological or synthetic nature. However, the comprehensive cellular response of bioprinted MCF7 breast cancer cells (BCC) or any other cells at the molecular level has not been published, yet it is crucial to determine whether bioprinted cancer models can potentially be used to predict drug efficacy, toxicity, and safety.

It has been widely suggested in the literature that bioprinting technology could lead to the pivotal discoveries of tissue engineered products which can be used for a range of clinical applications, e.g., skin grafting, tissue regeneration, cartilage repair, and others (Cui et al., 2012a, Yanez et al., 2014; Gudapati et al., 2016; Miri et al., 2019; Yerneni et al., 2019). However, this approach has not been used to develop tumor models *in vitro* for drug discovery. Recently Chen et al. and Phamduy et al. developed a bioprinting system where mass spectrometry was used in single printed cells. The authors (Phamduy et al., 2015) used laser direct-write cell bioprinting to bioprint MDA-MB-231 and MCF7s directly onto *ex vivo* rat mesentery tissue. They were able to monitor cell viability, proliferative and migratory properties and observed cell attachment and cell invasion within 2–5 days. Yet analyzing molecular and physiological changes in BP MCF7 BCCs has not been done and is long overdue. Here we report viability, apoptosis (programmed cell death), kinase phosphorylation, and RNA sequencing (RNA seq) analysis of BP MCF7 BCCs.

## Experimental Procedures

### Cell Culture

In this study, MCF7 (ATCC® HTB-22™) breast cancer cells were used for the *in vitro* experiments. Eagle's minimum essential medium (EMEM), supplemented with 0.01 mg/L Human recombinant insulin and 10% fetal bovine serum (referred to as media), trypsin 0.25% EDTA, and sterile phosphate-buffered saline (PBS) solution were used. Briefly, MCF7 cells were cultured per ATCC's cell protocol, a 75 cm flask with 8–10 ml of media were incubated in a humidified incubator maintained at 37°C with 5% CO_2_ until 80–90% confluency was reached cells were split and passaged to ensure cell stability.

### Bioprinting Process

In preparation for bioprinting, MCF7 cells growing around 80–90% of confluency were gently rinsed with PBS (to remove dead cells and debris) and detached with trypsin harvested and centrifuged at 800 rpm for 5 min. The supernatant was removed, and the cell pellet was next rinsed in PBS. Then the cells were counted with trypan blue and a hemocytometer. Modified inkjet cartridges adapted for a modified HP thermal inkjet printer were used to for bioprinting purposes (Wilson and Boland, 2003; De Maria et al., 2013). Next, 100 μL of PBS-cell solution (~1.6 × 10^6^ cells/mL—bioink) was added and printed into a tissue culture treated petri dishes (100 × 15 mm) or a Falcon, 96-well black/clear, tissue culture treated plate, flat bottom with lid. Following the bioprinting process, the cell viability analysis was conducted at 24 and 48 h utilizing two approaches: by using the Invitrogen™ Countess™ automated cell counter and through manual count by two different lab members using trypan blue and a hemocytometer. In these series of experiments, three independent measurements were accomplished, each performed in triplicate. Collected data are depicted as an average and standard deviation.

*Examination of the apoptosis/necrosis pathway via flow cytometer:* MCF7 Cells were bioprinted at a density of ~600,000 cells/dish in 6 ml of media. After 24 and 48 h post-bioprinting, cells were collected as above and double-stained by using the Annexin A5- FITC/propidium iodide (PI) staining kit, which is typically used to discern whether the cells are dying via apoptosis or necrosis pathway in the Beckman Coulter flow cytometer. The total percentage of apoptotic cells is depicted as the sum of both early and late stages of apoptosis (Annexin A5-FITC positives), whereas the cells stained only with PI, were considered as the necrotic cell population (Robles-Escajeda et al., 2016). Data acquisition and analysis were performed by using the Gallios flow cytometer (Gallios Beckman Coulter: Miami, FL) and the Kaluza software version 3.1. (Beckman Coulter) as previously detailed (Santiago-Vázquez et al., 2016).

### Stain Process

Morphological characteristics of the BP and MS MCF7 cells were evaluated with laser-confocal microscopy. Twenty-four hours post-bioprinting, cells were stained as explained elsewhere; (Iglesias-Figueroa et al., 2019) briefly, cells were fixed in 4% formalin for 20 min, washed and incubated in 0.1% v/v Tween 20 in PBS for 10 min at room temperature (RT), washed twice more with permeabilizing solution, incubated again in 200 μl of 5% w/v in bovine serum albumin (BSA; sigma) dissolved in tris-buffered saline solution containing 0.5% v/v Tween 20 for 1 h at RT on a rocker platform. The cells were next stained with the primary antibody Neu (sc-33684, dilution: 1:50) overnight in a rocker platform at 5°C. The cells were next washed three times with permeabilizing solution and a 1:50 v/v dilution of secondary antibody goat anti-mouse IgG conjugated with Alexa Fluor™ 568 (Invitrogen) was added and then incubated for 1 h on a rocker platform at RT. They were rinsed three more times with permeabilizing buffer and posteriorly they were co-stained with 0.165 μM of Phalloidin Alexa Fluor™ 488 (Invitrogen), and 5 μg/ml of 4′,6-diamidine-2′-phenylindole, dihydrochloride (DAPI, Invitrogen) for 1 h on a rocker platform; rinsed three more times with 0.1% v/v Tween 20 in PBS and leaving 200 μl in the wells at the end. Finally, using an inverted confocal-laser-scanning microscope (model LSM 700: Zeiss; New York, NY) assisted by the Zen 2009 software (Zeiss), to acquire high-quality digital images in three fluorescence channels (Alexa 568, Alexa 488, and DAPI). The enhanced contrast Plan-Neofluar 40x/1.3 oil immersion, differential interference contrast objective was used. The parameters to capture the high resolution images were consistently maintained; single-plane images were consecutively scanned for each fluorescence channel setting the pinhole at 1 Airy Unit.

### Phospho-MAPK Antibody Array (BP Cells) Process

The antibody array phospho-mitogen-activated protein kinase (phospho-MAPK) (R&D Systems #ARY002B) was used to analyze the phosphorylation levels of 26 kinases, which includes 9 MAPKs, ERK1/2, JNK1-3, and p28 isoforms in order to understand how the relative phosphorylation levels are affected by the bioprinting process. A collective sample of BP MCF7 BCC was harvested at 12 h, and 24 h post-bioprinting. Samples were processed as per the kit's protocol. Briefly, cells were solubilized in lysis buffer, diluted, mixed with detection antibodies, and incubated overnight in a rocking platform at 5°C with the phospho-MAPK array membranes. The membranes were washed the next day and detection reagents were applied. Upon completion of the reagent detection step, the iBright™ FL1000 Imaging System (Invitrogen Thermo Fisher Scientific) was used to develop the membranes. Microarray data was obtained by pixel density values, which were normalized, analyzed, and quality controlled by using Invitrogen™ iBright™ imaging software v.3.0 (Thermo Fisher).

### RNA Extraction and Sequencing

RNA sequencing was conducted in BP MCF7 BCCs at 2, 12, and 24 h post-bioprinting with the intention of identifying genes that were upregulated or downregulated by this process. Samples for RNA sequencing were prepared following the same bioprinting protocol as mentioned above. BP MCF7 BCCs were next gently detached with a cell sweeper to avoid exposure to influencing solutions, centrifuged at 800 rpm for 5 min and collected at 2, 12, and 24 h. RNA extraction was conducted for each sample with the PureLink RNA mini kit from Thermo Fisher and used per manufacturer's protocol. RNA concentration was evaluated with a Nano-drop 2000 spectrometer (Thermo Fisher Scientific). RNA Integrity Number equivalent (RIN^e^) was assessed for each sample and ranged from eight to ten. RNA seq data was analyzed for data summarization, normalization and quality control using Trimmomatic (version 0.36), Bowtie2 (version 2.2.5), and Cufflinks (version 2.2.1) (Langmead and Salzberg, 2012; Trapnell et al., 2012; Bolger et al., 2014). Differentially expressed genes were selected by using threshold values of >2 and <-2-fold change and a *q* ≤ 0.05. The *q-*value is used in genome-wide expression data, this statistical method is used to filter the proportion of false positives from a collection of *p* < 0.05.

STRING was used to map pathways of upregulated and downregulated differentially expressed genes. Cytoscape v.3.7.1., was next used to compare both network samples. Finally, network analysis to identify protein-protein interactions (edges or protein-protein connections) was extracted from Cytoscape. The number of connections or interactions are important parameters when targeting specific proteins associated with a disease. Genes with a >2-fold and <-2-fold (upregulated and downregulated genes) and a *q* < 0.05 were further classified by their gene ontology (GO) terminology. Upregulated and downregulated genes in the BP cells were divided into 3 groups, by their associated biological processes (bp), molecular functions (mf), and cellular components (cc). We also used STRING to create a protein network and compared the results of the MS to the BP MCF7 cells. We further mapped the protein pathways associated with genes expressed only in the BP cells. RNA and DNA information/analysis were compiled from: STRING, Panther, and Cytoscape 3.7.1 the following websites were also used to extract protein/gene connections: SMART, gene ontology, UniProt, and the Online Mendelian Inheritance in Man®.

### Statistical Analysis

Continuous variables for cell response to bioprinting were summarized using means and standard deviation (SD) of triplicate samples. In this experiment, 3 independent samples at 2 different time frames (24 and 48 h) were used to calculate viability and a one-way ANOVA analysis was conducted to compare the means for each time frame of the BP and manually seeded MCF7 BCCs, negative controls were also conducted. For the antibody array assay, data was centered, normalized, and clustered utilizing the iBright™ Analysis software version 3.0. All statistical analysis were completed using Minitab 18, IBM SPSS Statistics 25 and excel 2013.

## Results

### Post-bioprinting Cell Viability and Apoptosis

**Figure 1:**
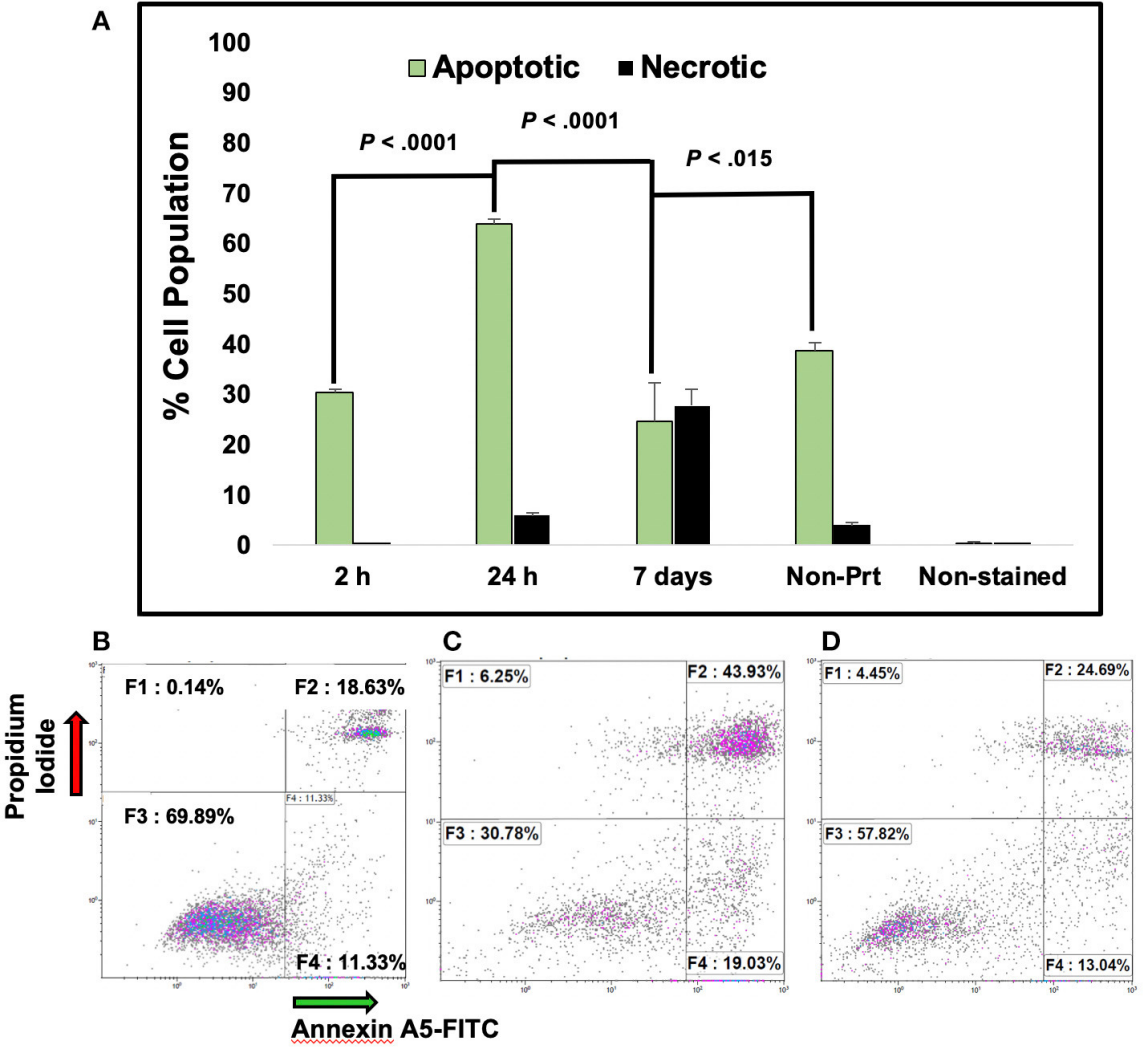
Percentage of Apoptotic MCF7 breast cancer cells post-bioprinting. The annexin A5-FITC kit was used for this analysis. Cells were collected post-bioprinting at 3 time frames, 2, 24 h, and 7 days Cells were BP in a modified HP thermal inkjet printer, onto a petri dish with EMEM, and the cells were incubated immediately. Graph **(A)** depicts the total percentage of apoptotic cell population is expressed as the sum of early and late apoptosis percentage (F2+F4), green bars. Dark bars depict necrotic cells, those are the cells stained with PI. Each bar represents the average of triplicates. Controls used were live cells stained, unstained, and dead cells (not shown). Error bars represent the standard deviations. **(B)** Dot plot graphs depict results at 2 h post-bioprinting. Graph **(C)** displays results at 24 h post-bioprinting. Graph **(D)** are the results from non-printed (manually seeded) MCF7s cells. *P*-values are from a two-tailed student *t*-test for independent tests (shown over the bars in **A**). 10,000 events were obtained per sample. The Kaluza Analysis v.1.3 software (Beckman Coulter) was used to extract the data and graphs.

The mean cell viability from the manual cell count process of the BP cells was 73.5% tested at 24 and 48 h and the average percentage of cell viability for MS (non-printed) cells was 98.6%. The average percentage cell viability from the automated cell count of the BP cells at 24 and 48 h was 76.7 and 72.8%, respectively. In the flow cytometer, samples collected at 2, 24 h and 7 days post-bioprinting, showed a percentage rate of live cells of 70, 30, and 47%, respectively. Apoptosis rates at these time frames were 30, 69, and 29%, respectively, and are shown in Figure 1A.

### Cell Morphology Results

**Figure 2:**
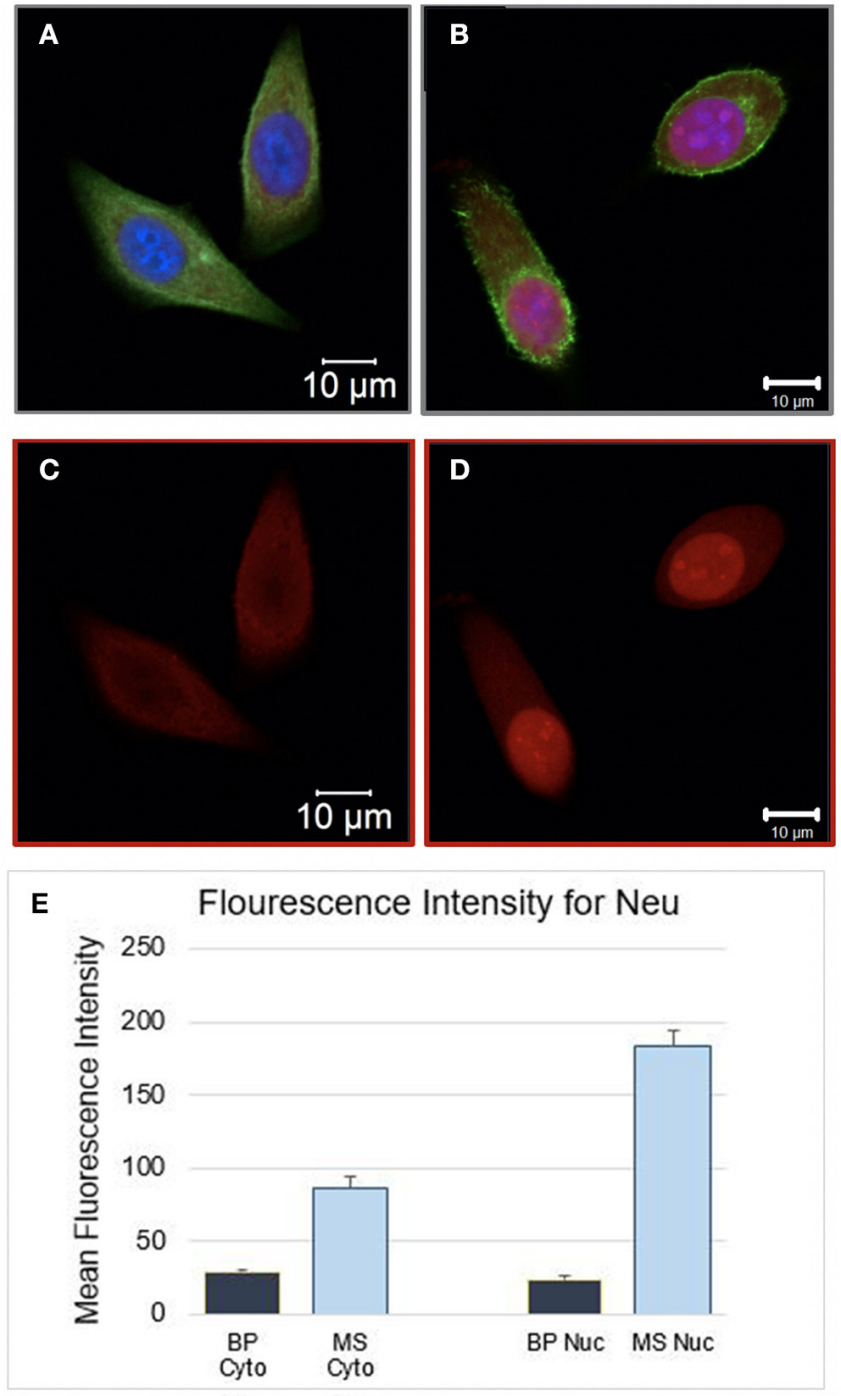
**(A)** Bioprinted MCF7 cells 24 h post-BP. Image showing in all three channels. **(B)** Manually Seeded MCF7 fixed at 24 h post seeding. **(C)** Bioprinted MCF7 Cells and **(D)** manually seeded MCF7 cells, single channel image, stained with Neu (sc-33684) primary antibody and goat IgG anti-mouse secondary conjugated with Alexa 568 channel. The same parameter settings were used for both cell samples. Fluorescent Intensity measurements were significantly different between the two samples, cytosol and nucleus were measured separately. In **(E)** Mean intensity measurements for the cytosol and the nucleus of the BP cells were 28.9 (1.6) and 24.3 (1.7), respectively. The mean intensity measurements for the cytosol and nucleus of MS cells were 87.0 (7.4) and 183.6 (9.8), respectively (BP Cyto = BP Cytosol, MS Cyto = Manually Seeded Cytosol, MS Nuc = Manually Seeded Nucleus, BP Nuc = BP Nucleus).

Comparing the morphology of both, BP and MS MCF7 cells, no major changes were observed between the two cell samples. Additionally, the fluorescence intensity of the Neu antibody was measured to quantify protein expression on both cells samples, the fluorescence intensity measurements were significantly higher by 3-fold in MS cells than in the BP cells (*p* < 0.001). Fluorescent parameter settings in that channel, Alexa 568, was the same for both samples. Visually, fluorescence intensity in MS cell samples was markedly more evident than BP cells as seen in Figure 2.

### Phospho-MAPK Array Results

**Figure 3:**
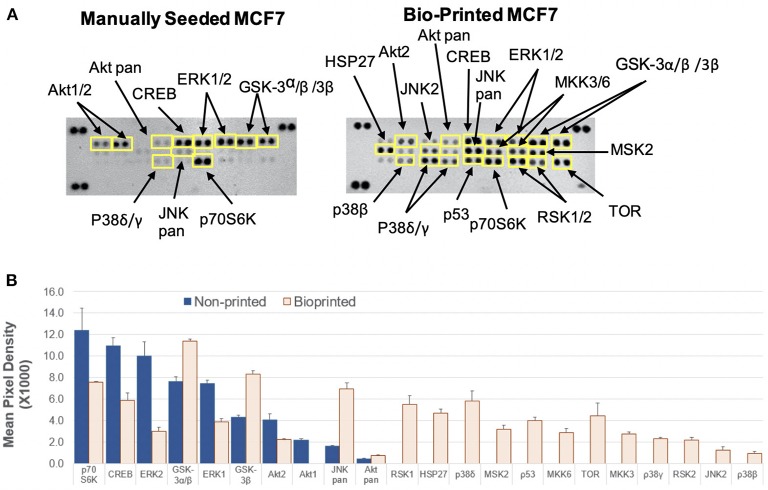
Activation of cellular kinases by thermal inkjet bioprinting. Chemiluminescent images in iBright FL1000 of a Proteome Profiler Human Phospho-MAPK Array (Catalog # ARY002B). **(A)** Membranes of manually seeded and BP MCF7 breast cancer cells. Membrane arrangement: LEFT = manually seeded (MS) MCF7 cells, RIGHT = BP MCF7. Signal for each kinase is represented by a pair of duplicate spots; three reference pairs are shown in three upper/lower corners. **(B)** Histogram profiles for selected analytes were generated by quantifying the mean spot pixel density exposure in the iBright FL1000 and Invitrogen™ iBright™ Analysis Software v.3.0. Kinases that show increased levels of phosphorylation are identified. Mean pixel density for the analytes is shown in the bar graphs. Twenty one kinases appeared phosphorylated in the BP samples, whereas 10 kinases showed in the manually seeded. Of the MS cells 6 kinases were strongly phosphorylated by >1.6-fold as compared to the BP cells.

**Figure 4:**
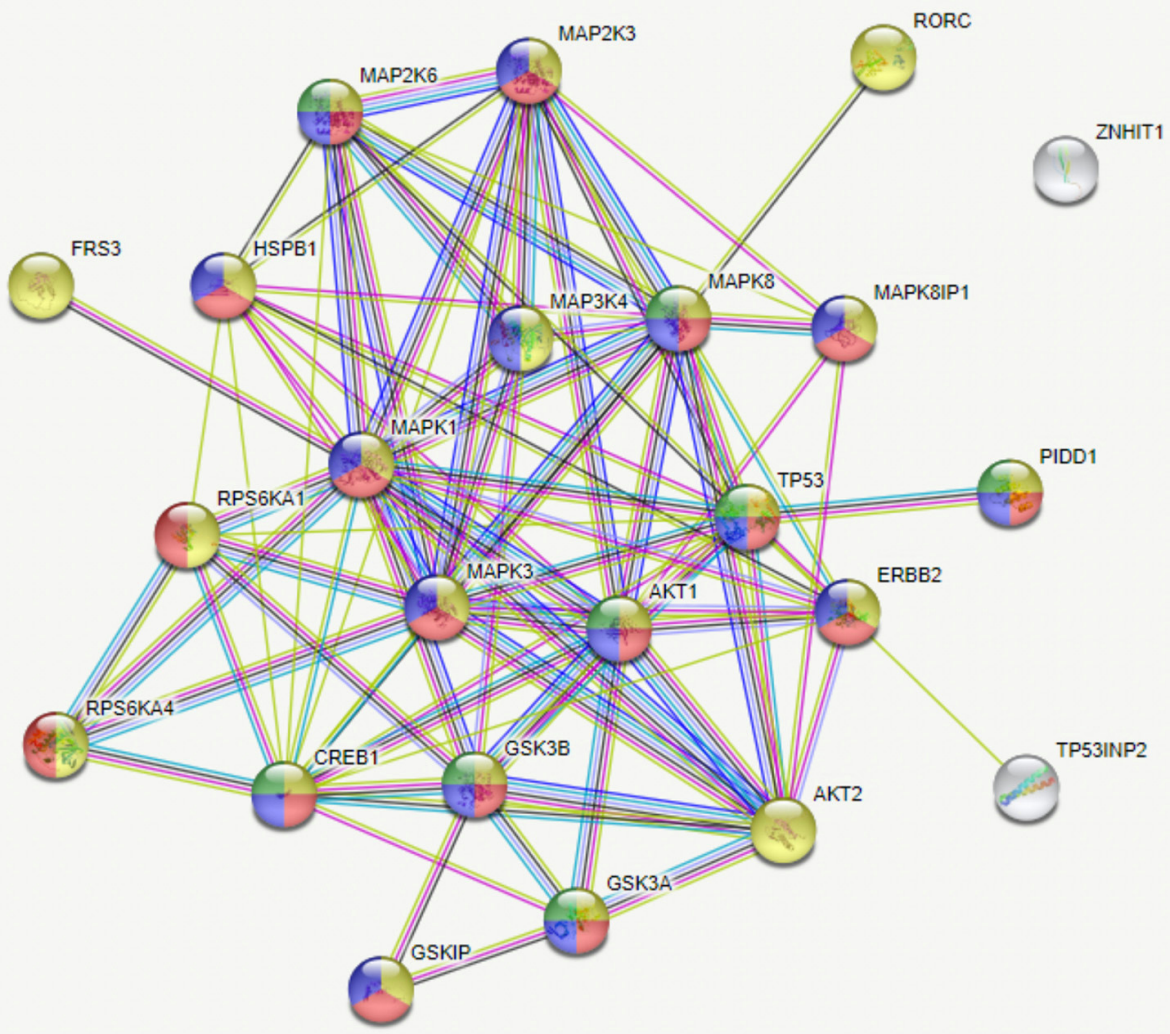
Network of analytes phosphorylated in BP MCF7 breast cancer cells. Functions selected in this network were regulator of apoptosis (green, 8), response to stress (red, 17), intracellular signal transduction (yellow, 21), and signal regulators (blue, 16). This network depicts functional interactions among BP BC predisposed genes. In this network of phosphorylated sites, there are significantly more interactions than expected (*p* ≤ 0.001).

**Figure 5:**
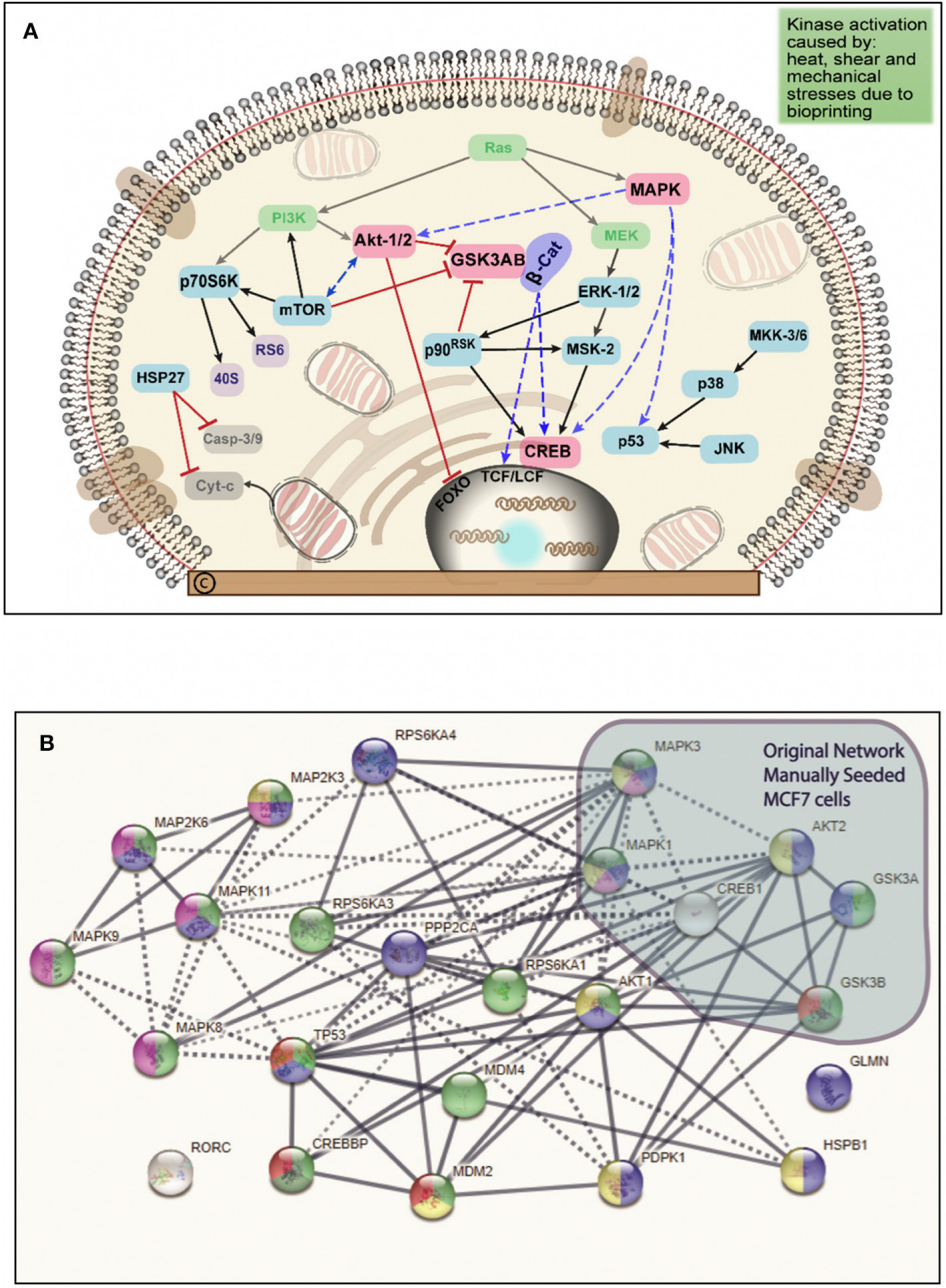
**(A)** Schematic summary of the intracellular pathways activated by bioprinting MCF7 cells. Kinases in green ovals not included in the phosphorylated kinases but they represent critical kinases, pink ovals represent overlapping kinases, present in both, manually seeded, and bioprinted cells. Blue ovals represent kinases phosphorylated only in BP cells. Red connector lines ending in a perpendicular line mean the originating kinase blocking signaling activity or deactivation. Representation of the critical kinases and their pathways in the bioprinted cells. **(B)** Network with links classified based on curated evidence in STRING. Biological functions selected are p53 binding process (red, 4), regulation of phosphorylation (blue, 14), stress activated protein kinase signaling cascade (pink, 7), regulation of cellular response to heat (dark green, 4), and cellular response to stress (light green, 16). These nodes have a significant number of interactions, as expected due to phosphorylated targets were extracted. The interaction score was set at 0.7 with k-mean clustering set at 5, thus only links that have a high confidence probability are displayed (*P* < 0.001). A total of 24 targets were observed from the phosphorylated targets in Bioprinted cells whereas for manually seeded cells, 6 phosphorylated sites were observed in this network. Despite the complexity of this network, we observed that MAPK1, TP53, CREB1, MAPK3, MAPK8, AKT1, HSPB1, AKT2, and MAP2K3 proteins display more than 10 protein-protein interactions.

The BP MCF7 cells phospho-MAPK array shown in Figure 3 revealed a total of 21 highly phosphorylated sites, whereas the MS cells revealed 11 phosphorylated sites. Of the phosphorylated targets in the MS cell samples, 6 analytes displayed 1.6-fold stronger phosphorylation levels than the BP cells (targets: p70S6 Kinase, CREB, ERK2, ERK1, Akt1, and Akt2), though no statistical significance was observed (*p* = 0.272). On the other hand, the BP MCF7s displayed 21 phosphorylated sites of which 12 did not show in the MS cells. These sites were: RSK1, HSP27, p38δ, p38β, MSK2, p53, MKK6, TOR, MKK3, p38γ, RSK2, and JNK2. Phosphorylated targets above the threshold were further investigated to associate targets with key biological processes, molecular functions, or the cellular components in the cells and to correlate with RNA sequencing results. In the network analysis shown in Figures 4, 5, a total of 24 targets were observed from the phosphorylated kinases in BP cells whereas for MS cells, 6 phosphorylated sites were observed in this network. This analysis showed MAPK1, CREB1, TP53, MAPK3, MAPK8, AKT1, HSPB1, MAP2K3, and AKT2 with >10 direct edges in the BP cell sample group.

### RNA Sequencing Results

RNA seq analysis conducted in BP MCF7 cells produced a total of 12,235 genes, of which 1,187 (9.7%) were significant (*q* ≤ 0.05). Using a cutoff value of a ±2-fold change there were 266 upregulated and 206 downregulated genes shown in Supplemental Information II. Furthermore, in the Log2 transformation of expression values, a total of 65 genes were significant (*q* ≤ 0.05), with five genes expressed only in the BP cells (NRN1L, LUCAT1, IL6, CCL26, and LOC401585). From the downregulated genes, every gene that showed in the MS cells also appeared in the BP cells. Of the five genes expressed in the BP cells, four gene ontologies (GO) in the molecular function were identified: protein binding, catalytic activity, molecular function regulator, and molecular transducer function (GO:0005488, GO:0098772, GO:0003824, and GO:0060089), four in the biological process: cellular response to stimulus, regulation, localization and processes (GO:0065007, GO:0009987, GO:0051179, and GO:0050896), and two in the cellular component ontology: the extracellular region and plasma membrane, which includes other external encapsulating systems such as the cell envelop (GO:0005623 and GO:0005576).

The following 4 genes, EFCAB11, FAM117B, FAM46B, and RBM43 were downregulated <-2-fold and the following 3 genes, GCLM, MOB3C, and VDR were upregulated >2-fold. No significant connections were found among those genes. EFCAB11 contains 11 nodes and 11 edges, though no statistical significance was observed, with an interaction score set at 0.4. Recent publications have associated this gene with hepatocellular carcinoma. Of the genes that were upregulated and have more than 10 protein to protein interactions are: CYP1A1, IL6, UGT1A6, EGFR, and CYP1B1 (see Table 1). Cytochrome P450 1A1 (CYP1A1) was upregulated by more than 300-fold and CYP1B1 by 10-fold in the BP cells. Cytochromes P450 are a group of enzymes involved in catalyzing endogenous substrates, including steroids, fatty acids, xenobiotics, and others (Spink et al., 1998). These CYP enzymes are accountable for DNA damage, which leads to tumor inception (Mikhailova et al., 2005). On the other hand, downregulated genes in BP cells with ≥10 protein interactions are: TP53, FOS, JUN, EGR1, HIST2H2AC, and FOSB (see Table 2). TP53, aka tumor suppressor gene, is further repressed in these cells; this explains the added resolute characteristics hence death evasion (Campbell et al., 2019). A Complete list of gene analysis is available in Supplemental Information II.

**Table 1:** List of genes extracted from the upregulated genes (>2 fold) in the bioprinted MCF7 cells RNA seq analysis, this table contains only genes with ≥5 number of edges, network diagram depicted in Supplement Information I, Figure S2.

**GENES**	**Closeness centrality**	**Degree**	**Number of directed edges**
CYP1A1	0.22222222	14	14
IL6	0.34024896	13	13
UGT1A6	0.22162162	13	13
EGFR	0.33744856	12	12
CYP1B1	0.22102426	12	12
UGT1A3	0.18594104	9	9
UGT1A4	0.18594104	9	9
UGT1A10	0.18594104	9	9
UGT1A8	0.18594104	9	9
UGT1A9	0.18594104	9	9
UGT1A1	0.18594104	9	9
UGT1A7	0.18594104	9	9
PSMD1	0.22465753	7	7
AKR1C3	0.18594104	6	6
NQO1	0.25867508	6	6
HMOX1	0.29927007	6	6
ITGAV	0.23563218	6	6
CD44	0.27152318	6	6
PSME4	0.22343324	5	5
ICAM1	0.26537217	5	5
PPARG	0.32156863	5	5

*The number of directed edges represent the number of interactions between the listed protein and other proteins*.

**Table 2:** List of genes extracted from the downregulated genes (<-2 fold) in the bioprinted MCF7 cells RNA seq analysis, this table contains only genes with ≥5 number of edges, network diagram depicted in Supplement Information I, Figure S5.

**GENE**	**Betweenness centrality**	**Closeness centrality**	**Degree**	**Number of directed edges**
TP53	0.62411488	0.50420168	20	20
FOS	0.27122411	0.46875	15	15
JUN	0.10417137	0.45112782	12	12
EGR1	0.12483051	0.43478261	12	12
HIST2H2AC	0.16229755	0.37037037	11	11
FOSB	0.03096045	0.37735849	10	10
MYC	0.12	0.42253521	9	9
EGR2	0.03934087	0.36144578	8	8
KIF20A	0.08163842	0.28037383	6	6
ATF3	0.01900188	0.4	5	5
HMMR	0.04887006	0.27906977	5	5
NR4A1	0.00951977	0.40268456	5	5
CTGF	0.09722222	0.39473684	5	5

*The number of directed edges represent the number of interactions between the listed protein and other proteins*.

## Discussion

The present study evaluated morphology, viability, apoptosis, and necrosis, phospho-MAPK array, and RNA seq analysis of BP MCF7 BCCs. The cellular morphology of BP cells was compared to the MS cells. The BP cells appeared to have blebs, and non-spherical and distorted bulky features when compared to the MS cells (see images in Supplemental Information I). These protrusions may be caused by shear and mechanical stresses during bioprinting. Wickmann et al. identified blebbing as one of the distinctive characteristic of apoptosis (Wickman et al., 2013). This apoptotic morphological feature was confirmed by performing the Annexin A5-FITC necrosis/apoptosis assay, where after several hours following bioprinting, a number of cells appeared in the early and late apoptosis phase (Figure 1).

BP cells were interrogated with the neu antibody, neu is a glycoprotein expressed in ~30% of the cancers, yet not highly expressed in MCF7 cells. Neu (HER2) was probed in the BP and MS MCF7s to test the hypothesis that bioprinting would stimulate expression of the HER2/neu protein. However, results were contradicting, with MS cells showing significantly higher neu expression (Figure 2, *p* < 0.0001. This response is attributed to induced stress due to bioprinting, thus decreasing neu protein synthesis in the entire cell. We further compared the morphology of bioprinted and manually seeded cells and observed that the BP cells appeared smaller and flatter.

### Viability, Apoptosis, Necrosis

Viability results were comparable to other published results which indicate a viability greater than 70% (Xu et al., 2013; Kolesky et al., 2016) but in some systems it can be as high as 90–98% (Cui et al., 2012b; Xu et al., 2013). Twenty-four hours post bioprinting (Ma et al., 2015). Chang et al., reported that BP HepG2 cells' viability in a multi-syringe nozzle ranged between 70 and 80% (Chang et al., 2008). Though, in that study, the bioink used to bioprint cells was sodium alginate crosslinked with CaCl_2_. In our experiment, the viability of the BP cells ranged from 60 to 87% tested at both, 24 and 48 h. Additionally, there were no media replenishments, which may have inflated viabilities in other studies. In brief, during the thermal BP process, cells are discharged from the modified print cartridge at ~1 m/s (Kim et al., 2016), they pass through a heated filament where the temperature is raised close to 300°C for a few microseconds (Calvert, 2001; Roth et al., 2004). Therefore, BP cells in a thermal inkjet printer are briefly heated as they pass through the printing filament before landing in a petri dish with pre-incubated cell media at a pH of 7.2 (Mironov et al., 2003; Roth et al., 2004; Singh et al., 2010). It is worth noting that during cell count preparation, most of the cells that did not survive bioprinting may have been discarded during the cell count preparation process because those cells were already detached from the petri dish, thus when centrifuged and supernatant was gently decanted some of the floating cells might have been removed, leaving mostly live cells in the pellet. Previous reports of BP cells have indicated viability rates ranging from 70 to 90% (Cui et al., 2012a; Peng et al., 2017).

Moroi et al. observed that tumor cells exposed to a 43°C water bath for 30 min quickly entered apoptosis depending on the localization of the tumor cells (Moroi et al., 1996). In addition, they observed that necrosis did not change immediately but it gradually increased within 3–6 h (Moroi et al., 1996). Measurements of results from the Annexin A5 analysis at 2 h post-bioprinting, resulted in an apoptotic rate of 30.5% with a necrotic rate of 0.1%. The apoptotic rate increased by 15% and the necrotic rate by 5.9% at 24 h following bioprinting. The percentage of live cells in this assay was 69 and 30% at 2 and 24 h, respectively, suggesting that bioprinting causes damage to the cells. On average, 12 and 19% of the BP cells evaluated at 2 and 24 h post-bioprinting appeared in the early apoptotic cycle. As sampling collection time was increased post-bioprinting, a 2.4-fold increase in the late apoptosis stage was observed 24 h later and a 1.15-fold increase 48 h. In another study, Catros et al. reported a mortality rate of endothelial cells embedded in sodium alginate solution BP with a laser-assisted bioprinter between 60 and 37%, which was dependent upon the thickness of the Matrigel™ bioink used and the laser energy applied, which varied between 20 to 100 μm and from 8 to 24 μJ, respectively (Catros et al., 2011). They observed a lower mortality rate in thicker gels that were 80–100 μm thick when higher laser energy was applied (Catros et al., 2011). However, the actual mortality rate was not directly measured, it was concluded from observed images. Bioprinting may be causing thermal and mechanical stress to the cells resulting in a high number of stressed and weak cells that when subjected to the Annexin A5-FITC assay, became apoptotic, instead of fully convalescing. This is important when considering drug tests, BP cells shall be allowed recovery time with at least one media change prior to testing any drugs (Campbell et al., 2019).

### Phospho MAPK Array—Phosphorylated Analytes in BP MC7 BCCs

*Glycogen synthase kinase-3* (GSK3 or GSK-3β), regulated by the Ras signaling pathway, stimulates cell proliferation (Milisav et al., 2017). GSK-3α, a Ser/Thr kinase, was identified as a deactivator of glycogen synthase and a regulator of other cellular functions, like cancer cell survival and proliferation (Ugolkov et al., 2016). In another arrangement, when GSK-3β is active, it phosphorylates β-catenin causing it to degrade (Brembeck et al., 2006; Weinberg, 2013). If however, GSK-3β is deactivated, β-catenin translocates to the nucleus where it binds Tcf/Lft Transcription Factors (TF) and stimulates expression of various genes related to cell proliferation amongst others (Weinberg, 2013). GSK-3β is deactivated via phosphorylation by the Akt/PKB pathway; this inhibition prevents cells from entering apoptosis (Brembeck et al., 2006; Weinberg, 2013). Inhibition of drug induced apoptosis may be triggered by GSK-3β and therefore, the bioprinted cell model may potentially mimic this process.

GSK-3 works as a multifunctional downstream switch that influences the output of several signaling pathways (Woodgett, 2005; Arcaro and Guerreiro, 2007) and dysregulated GSK-3 has been implicated in cancer. GSK-3α/β is highly phosphorylated in both cell sample conditions, yet in the BP cells, phosphorylation was 1.6-fold higher than in MS cells. GSK3α/β are encoded by GSK3A and GSK3B genes, respectively. In the RNA seq analysis, expression of this gene was upregulated in the BP cells by 1.2-fold (*q* = 0.1, 0.5) for both. Through the protein-protein network analysis we observed that this protein contains 9 interactions, and mutated GSK-3 can activate or de-activate other signaling pathways and collectively stimulate cancer progression.

*Heat shock proteins* (HSPs) have been identified to belong to a large family of stress response proteins and they function as molecular chaperones by assisting with the folding/unfolding of other cellular proteins and keeping substrate aggregation (Kato et al., 1994). HSPs are normally expressed at low levels but under heat shock they are highly phosphorylated (Rogalla et al., 1999; Almeida-Souza et al., 2010). Elevated levels of HSPs has been found in ischemia/reperfusion, cancer, and have been linked to cancer angiogenesis (Kato et al., 1994; Vos et al., 2009). HSP27 also functions as an anti-apoptotic molecule, regulating apoptosis through direct interaction of key components of the apoptotic pathway, this occurs in cooperation with HSP70 (Bryantsev et al., 2007; Vos et al., 2009). Phosphorylation for this analyte was observed in BP cells only, and it was not shown in MS cells. In the RNA seq analysis of the MCF7 cells, expression levels is higher than in MS MCF7 cells although no statistical significance was found (*q* = 0.37). Thermal inkjet bioprinting exposes cells to heat pulses during the bioprinting process, which would explain the high phosphorylation levels found in surviving BP cells (Mironov et al., 2003; Xu et al., 2013). This confirms one of our hypotheses that thermally bioprinted BCC stimulate HSPs, in this case HSP27. Increased levels of heat shock proteins may trigger a cell survival/resistance mechanism in cancer cells. Additionally, HSPs activate the angiogenic pathway that leads to vascular endothelial growth factor (VGEF) expression, vessel formation, and accelerated tumor formation in host tissues (Chatterjee et al., 2018; Plimpton et al., 2018; Solis et al., 2019).

*Jun N-terminal Kinases* (JNKs) are small proteins, 45–55 kDa, and product of three genes which, through different splicing, create up to 10 isoforms (Alvarez et al., 1991; Hibi et al., 1993). When activated, JNKs translocate to the nucleus where they regulate the process of various transcription factors, including the activator protein 1 (AP1) (Okazaki and Sagata, 1995; Weinberg, 2013). AP1 is implicated in antiestrogen resistance in breast cancer, which results in poor therapy response to hormonal therapy. This kinase has also been implicated in several biological processes such as cell migration, differentiation, proliferation, transformation, and apoptosis (Dai et al., 2000; Weinberg, 2013). It has been shown that environmental stresses, like heat shock and osmotic shock can activate this pathway (Kurokawa et al., 2000), which is suspected to be the case in BP MCF7s. Phosphorylation levels of JNK in BP cells was 4.4-fold higher than in the MS cells, even though expression levels of this gene changed only slightly.

*Ribosomal S6 kinase* (RSK1) is a broadly expressed component of the RSK consort of growth factor-regulated ser/thr kinases (Robinson and Cobb, 1997), which stimulate cell proliferation and differentiation (Hu et al., 2004). RSK1/2 appeared highly phosphorylated in BP cancer cells but not in MS cells (see Figure 3). RPS6KA1 is highly upregulated in BP MCF7 cells by 1.6-fold compared to MS cells, and a statistical significance was observed (*q* = 0.001). This suggests that MCF7 cells surviving the bioprinting process may become more resilient and this characteristic makes their use as *in vitro* models a great option for use during drug discovery.

*P38* MAPKs are stimulated by proinflammatory cytokines, environmental stresses, hypoxia, and osmotic shock (Sayed et al., 2000; Tamura et al., 2000). Once stimulated, p38α phosphorylates several targets, which may act as tumor suppressors and the activation of those is important in cell proliferation and cell survival. Several compounds that inhibit p38α continue to be screened as potential anti-cancer therapies (Igea and Nebreda, 2015). P38α activation has been linked to tumor cell survival of chemotherapeutic treatments. In BP MCF7 cells, p38 was highly phosphorylated whereas in MS cells no phosphorylation was observed, while the expression levels in RNA seq were not significantly higher. The activation of p38 by bioprinting is a process that shows promise when used in tumor models as an anti p38 drug candidate.

*MSK2* is activated in stress-related signaling by P38a/MAPK (Pierrat et al., 1998) and is phosphorylated in BP cells but inactivated in MS cells. Cancer cells are known to metabolize large amounts of their glucose through the glycolysis cycle, rather than the citric cycle meaning that activation of MSK2 in the BP cells may assist with regulation of glycogen metabolism that promotes cell survival.

### Phosphorylated Sites in Both Manually Seeded and Bioprinted MCF7 BCCs

Seventy kilodalton ribosomal protein S6 kinase (p70S6) controls the activation of cell growth and is generally expressed in adult human tissues (Ferrari and Thomas, 1994; Cross et al., 1995). This activity increases during the G1 phase by 20-fold when released from the G0 phase of the cell cycle (Edelmann et al., 1996; Knowlton et al., 2011). P70S6K is highly phosphorylated in MS cells; however, phosphorylation levels decrease by 0.6-fold in BP MCF7 cells which suggest cell cycle arrest at G0. This explains low cell survivability levels post-bioprinting.

*The cyclic AMP Response Element-Binding Protein* (CREB) is part of the bZIP family of TFs. CREB is associated with cancer growth and poor clinical outcomes in several types of cancer, including breast cancer (Son et al., 2010). In MS cells, CREB and extracellular signal-regulated kinases 1/2 (ERK1/2) were constitutively phosphorylated by 2 and 3.5-fold, respectively, which is higher than in BP MCF7 BCCs but the gene expression levels do not differ significantly. The lower phosphorylation levels in the BP cells suggests that CREB may be regulating cell cycle progression in the MS cells yet not the BP cells; however, this could mean that CREB may be inactivated temporarily in the BP cells yet when these cells are allowed to recuperate from the bioprinting process, CREB levels may rise again to levels typical of cancer cells.

*p53* is known as a tumor suppressor protein, and it has been found mutated in many types of tumors (Bond et al., 2005; Vaseva et al., 2012). A number of events can stimulate increased p53 levels, such as exposure to acidic environments or to nitrous oxide, low oxygen tension, lack of an intracellular group of nucleotides, blockage of DNA or RNA synthesis and other insults that may occur in the cells (Ghosh et al., 2004; Vaseva et al., 2012). P53 phosphorylation levels were high in the BP cells however, it was completely deactivated in the MS cells. This suggests that p53 is highly dysregulated in these cells, yet it may also suggest that p53 could have been activated through the JNK signaling pathway as described above. However, in the RNA seq expression analysis, P53 were two-fold higher (*q* = 0.001) in the MS cells than in the BP cells, suggesting that p53 is further suppressed in the BP cancer cells, though the phosphorylation of p53 remains unknown at this point. It may be possible that p53 is deactivated in BP cells indirectly via the Akt signaling cascade.

*Mitogen Activated Protein Kinase Kinase 6* (AP2K6 or MKK6) has a critical role in the MAPK signal transduction pathway (Raingeaud et al., 1996), as it can activate p38 (Moriguchi et al., 1996; Raingeaud et al., 1996; Stein et al., 1996) in which then leads toward the specific activation of transcription factors, such as activating transcription factor 2 (ATF2) and ETS-like 1 (Elk1) (Goedert et al., 1997). ATF2 dysfunction has been linked to cancer metastasis (Watson et al., 2017) These proteins are activated by several insults, such as heat shock, UV rays, osmotic shock, cellular DNA damage and their regulation remains obscure (Goedert et al., 1997). The phosphorylation level of MKK6/MKK3 was observed in the BP cells yet it was not seen in the MS MCF7 cells. The MAP2K3 gene showed no statistical significance in expressions but MAP2K6 expression levels were significantly lower (0.35-fold) than in MS cells (*q* = 0.027). Hyper-phosphorylation of these kinases in BP cells is attributed to the mechanical, shear, and heat traumas triggered by bioprinting; this feature may be beneficial for use in tumor models due to the high number of phosphorylated sites, which could mimic *in vivo* stresses.

*Akts* participate in cell survival and proliferation, cycle progression, vesicle trafficking, glucose transport, metabolism, and biological processes (Noguchi et al., 2014; Ruiz-Medina et al., 2019). Akt's activation is believed to have a key function in tumorigenesis (Martini et al., 2014). Akt1 and Akt2 appeared phosphorylated in MS MCF7 cells. Phosphorylation of Akt1 is not observed in the BP cells. However, phosphorylation of Akt2 was 1.7-fold higher in the MS BCCs. Lower phosphorylation levels in BP cells suggest that its role as glucose uptake regulator is limited temporarily due to the effect of bioprinting. Akts, aka proto oncoproteins, have several downstream effects when activated, two of which are the binding of B-cell lymphoma 2, BCL2-associated X (BAX) protein, producing an anti-apoptotic effect when BAX blocks the mitochondria. Another downstream effect is protein synthesis via Rheb, resulting in the activation of mTOR thus interacting and activating S6K to initiate mRNA translation (Tabatabaian et al., 2010).

*The extracellular signal-regulated kinases 1/2* (ERK1/2), once phosphorylated, can regulate several cellular processes such as protein synthesis, chromatin remodeling, and transcription (Weinberg, 2013). Mutations in proteins along the ERK pathway are common in cancers. In MCF7 cells, the ERK pathway is activated by estradiol (Robinson and Cobb, 1997). While ERK1/2s were highly phosphorylated in MS cells, the phosphorylation levels in BP cells is lower by 0.3 and 0.5-fold and the RNA expression levels for both ERK1 and ERK2 are not significantly different. Lower phosphorylation levels in the BP cells indicate some signaling is still evident and this may be a transitional event for ERK1/2 can be activated via the Akt/MEK pathway.

*Mammalian Target of Rapamycin* (mTOR) a ser/thr protein kinase acts as the main regulator related to energy and stress signaling, cellular metabolism, growth factors and nutrients, growth and survival in response to hormones, autophagy and cytoskeletal re-organization (Kim et al., 2002; Park et al., 2002; Inoki et al., 2003; Brugarolas et al., 2004). mTOR was found to prevent autophagy through phosphorylation both directly through the phosphorylation of ULK12 and indirectly through DAP1 (Koren et al., 2010). This analyte, is phosphorylated in the BP MCF7 cells but not in the MS MCF7 cells, which suggests that newly bioprinted cells may be inducing cell proliferation. Expression levels for the TOR1A and mTOR were not statistically significant. Getting to know the phosphorylated pathways of bioprinted cancer cells at the molecular level is critical when considering this process in order to use these tumor models in drug discovery.

### RNA Sequence Analysis

For decades, preclinical and clinical evidence have shown that cancer cells exhibit significantly higher sensitivity to hyperthermia than normal cells. Intratumoral cell temperatures range from 42 to 45°C (Habash et al., 2006). A cellular exposure of 45°C for 30 min specifically, altered the expression of key mitotic regulators and altered G2/M phase progression in breast cancer cells, including the MCF7 cell line (Amaya et al., 2014). Thus through RNA seq analysis, we sought to explore the differences in gene expression of the manually seeded and bioprinted MCF7 cells as the bioprinting process further exposes cells to high levels of heat.

The non-protein coding gene Lung Cancer Associated Transcript 1 (LUCAT1) was among the genes found differentially and significantly overexpressed in the BP cells as compared to the MS MCF7 cells; yet expression levels found in breast cancer stem cells (BCSCs) is higher than in normal BCCs (Zheng et al., 2019). Upregulated LUCAT1 has been implicated with BCC proliferation and shorter overall survival and progression-free survival (Yoon et al., 2018). LUCAT1 is a member of the long intervening noncoding RNAs (lincRNAs) class that seems to function downstream on the nuclear factor erythroid 2–related factor 2 (NRF2) (Tonelli et al., 2018). NRF2 controls gene expression that regulates oxidative stress protection in airway epithelial cells (Thai et al., 2013). Thus, it is likely that the LUCAT1's overexpression is an important contribution to the augmented resistance to death of the BP MCF7 cells *via* NRF2, which is a developing regulator of cellular resistance to oxidants (Ma, 2013). LUCAT1, expressed only in BP MCF7 BCCs, suggests that bioprinting may be differentially selecting more specifically BCSCs. It is also possible that the genes associated with stem cell phenotype are already expressed in few of the cells beforehand and when the cells were bioprinted, it induced cell proliferation of more cells with the stem cell phenotype. LUCAT1 has been identified as a potential target for drug discovery (Zheng et al., 2019).

The C-C motif Chemokine Ligand 26 (CCL26) was also found to be overexpressed in the BP MCF7 cells, as compared to the MS cells. CCL26, also known as Eotaxin-3, is a chemotactic cytokine for eosinophils and basophils and exerts its effect by binding to its receptor CCR3 (Butterfield et al., 1993; Kitaura et al., 1996; Shinkai et al., 1999). It was reported that CCL26 is regulating expression of cancer-associated genes during airway inflammation (Marijani et al., 2011). Furthermore, the upregulation of this gene, as well as CCL2, IL6, and LOXL2, has been implicated as part of the effects of cancer-associated fibroblasts in promoting progression of hepatocellular carcinoma cells (Lin et al., 2012). Thus, the CCL26-CCR3 ligand-receptor structure appears to be involved in inflammatory processes and it is upregulated in cancer progression, which highlights the suitability as an *in vitro* model for anti-cancer drug discovery initiatives.

Of the genes that were downregulated in the BP MCF7s, in the molecular function ontology EF-hand calcium-binding domain-containing protein 11 (EFCAB11) is a protein coding gene and it has been implicated in hepatocellular carcinoma, and FAM117B (under biological process) has been found to negatively regulate activation-induced death in T-cells and to respond to osmotic stress. Upregulated, the glutamate-cysteine ligase modifier (GCLM) subunit is linked to apoptotic mitochondrial changes and it is a negative regulator of the extrinsic signaling pathway. This pathway utilizes a ligand binding process to induce apoptosis, which initiates in the extracellular milieu (Weinberg, 2013).

Under normal circumstances, cancer cells can bypass the *in vivo* removal tactic of the immune system by implementing diverse strategies such as evasion of apoptosis (Kobayashi et al., 1993). Those cellular eluding mechanisms include mutations that provide the ability to counteract the immune system, which fails to detect and eliminate. Cancer cells acquire the ability to undergo rapid proliferation without regard to the normal homeostatic system; uncontrolled proliferation is a distinctive characteristic of cancer development and progression (metastasis) (Feitelson et al., 2015). Furthermore, RNA seq analysis performed in bioprinted MCF7 cells provided significant information by identifying genes involved in response to the bioprinting process. These data clearly demonstrated that thermal inkjet bioprinting is triggering significant number of gene alterations that could potentially be used for target drug discovery.

We believe that bioprinting may stimulate other conditions like exacerbating pathways implicated in drug immunity, cell motility, proliferation, survival, and differentiation such as the expression of NRN1L, LUCAT1, IL6, and CCL26, which have been implicated with numerous diseases. This underscores the need to use these thermal inkjet bioprinting tumor models to simulate *in vivo* conditions where, in some cases, tumors develop immunity to chemotherapeutic drugs. Additional to these mutations, we have observed that cancer cells surviving the bioprinting process, elicit further phenotypic changes, which we believe makes them even more resistant to cancer drugs. Thus we are proposing to use bioprinted cancer cells, which become resilient and hard to kill, to discover a new class of more efficacious and potent cancer drugs. Collectively, these findings contribute with relevant information to affirm that this BP model can mimic a novel tumor model that should be studied further and applied in preclinical studies.

## Conclusion

Bioprinted MCF7 cells showed increased levels of phosphorylation in analytes that have been identified as key players in activating critical pathways that when dysregulated, are associated with biological aggressive oncogenic properties. Results of bioprinted MCF7 BCC at the physiological and molecular levels were evaluated at different time frames following bioprinting in a modified thermal inkjet printer. Despite losing up to 37% of the cell population 24 h post bioprinting, it is evident that this process induces ample phosphorylation at several critical sites. Several key signaling cascades were activated, as was observed in the phosphorylation of MKK6/MKK3 and RSK1/2, which have been implicated with cancer metastasis. Collectively, GSK-3α/β, ERK1, Akt1/2, JNK, RSK1/2, HSP27, p38, MSK2, p53, MKK3/6, and TOR when mutated or hyper-phosphorylated are implicated in biologically aggressive behaviors which could be used to develop an *in vitro* tumor model that could be used to explore drug discovery. Unregulated levels of heat shock proteins may also be triggering a cell survival mechanism in cancer cells like a conglomeration of receptors in the ECM. In this experiment, bioprinted MCF7 cells showed increased levels of chaperone protein HSP27. It is possible that bioprinting may be stimulating an increased amount of ligand-independent receptors in the cell surface. This occurrence may cause receptor collisions which lead to receptor dimerization thereby making them more sensitive in overexpressed genes associated with cellular resistance to intrinsic biological processes and cellular functions.

The RNA seq analysis of BP MCF7 cells indicates that BP MCF7 cells may be causing several mutations which enable cells to become more robust encouraging MCF7 cells to activate key kinases implicated in cancer development, proliferation, and metastasis. Furthermore, through the phospho-MAPK array, we confirmed that bioprinting activates signaling pathways associated with cellular response to apoptosis, mitosis, cell migration, transcription, and other cellular functions. Additionally, it may also be triggering other undiscovered functions in the BP BCCs, such as activation of critical pathways implicated in drug immunity or inducing cell motility, proliferation, survival, and differentiation. Moreover, these data suggest that thermal inkjet bioprinting is stimulating large scale gene alterations that could potentially be used with autologous drug tests to confirm drug efficacy prior to initiating cancer therapy. Insights into the cell response after bioprinting have demonstrated that BP cells can potentially improve the *in vitro* models for drug discovery.

## Data Availability Statement

The datasets generated for this study are available on request to the corresponding author.

## Author Contributions

AC, AV-R, and TB conceived and designed the study. AC, JM, and DG collected the data. AC, JM, AV-R, and TB analyzed and interpreted the data. AC, JM, AV-R, and DG wrote the manuscript. TB wrote parts of the manuscript and edited it to the final version.

### Conflict of Interest

The authors declare that the research was conducted in the absence of any commercial or financial relationships that could be construed as a potential conflict of interest.
