# Supplementary material for: Thermal Bioprinting Causes Ample Alterations of Expression of LUCAT1, IL6, CCL26, and NRN1L Genes and Massive Phosphorylation of Critical Oncogenic Drug Resistance Pathways in Breast Cancer Cells
Source: Front Bioeng Biotechnol. 2020 Feb 21;8:82. doi: 10.3389/fbioe.2020.00082 (PMC7047130; doi:10.3389/fbioe.2020.00082)
Supplement: Supplementary file 1 [file Image_1.pdf]

frontiers in Bioengineering and Biotechnology

Thermal Bioprinting Causes Ample Alterations of Expression of LUCAT1, IL6, CCL26 and NRN1L Genes and Massive Phosphorylation of Critical Oncogenic Drug Resistance Pathways in Breast Cancer Cells

Aleli Campbell[1], Jonathon E. Mohl[3], Denisse A. Gutierrez[2], Armando Varela-Ramirez[2], Thomas Boland[1*]

1. Metallurgical, Materials and Biomedical Engineering, University of Texas at El Paso, El Paso, TX. USA.
2. Border Biomedical Research Center, Department of Biological Sciences, University of Texas at El Paso, El Paso, TX.USA.
3. Department of Mathematical Sciences and Border Biomedical Research Center, University of Texas at El Paso, El Paso, TX. USA.

* Corresponding author
500 W. University Ave
El Paso, TX 79968
tboland@utep.edu

Supplemental Information - Part I

List of Figures

Figure S1. Network of analytes phosphorylated in BP MCF7 breast cancer cells………………………………………………………………………………...…3
Figure S2. Network diagram of upregulated genes in the RNA seq analysis of bioprinted MCF7 BCCs, protein-protein network……………………………..……4
Figure S3. Bar graphs depicting frequency of the different molecular functions or sites associated with the GO terms in the differentially expressed genes (upregulated)………………………………………………………………………..…….5
Figure S4. Bar graphs depicting frequency of the different molecular functions or sites associated with the GO terms in the differentially expressed genes (upregulated)………………………………………………………………………..…….6
Figure S5. Network diagram of downregulated genes in RNA seq analysis of bioprinted MCF7 BCCs, protein-protein network……………………………..……7
Figure S6. Bar graphs depicting frequency of the different molecular functions or sites associated with the GO terms in the differentially expressed genes (downregulated)……………………………………………………………….……….. 8

Figure S7. Bioprinted MCF7 breast cancer cells stained with Neu, Phalloidin (green) and DAPI. (Brightness settings untouched)……………………………9
Figure S8. Bioprinted MCF7 breast cancer cells stained with Neu, Phalloidin (green) and DAPI. (Brightness settings untouched)……………………………10
Figure S9. Bioprinted MCF7 breast cancer cells stained with Neu, Phalloidin (green) and DAPI. (Brightness settings untouched)……………………………11
Figure S10. Bioprinted MCF7 breast cancer cells stained with Neu, Phalloidin (green) and DAPI. (Brightness settings slightly retouched)…………………..12
Figure S11. Bioprinted MCF7 breast cancer cells stained with Neu, Phalloidin (green) and DAPI. (Brightness settings untouched)……………………………13
Figure S12. Bioprinted MCF7 breast cancer cells stained with Neu, Phalloidin (green) and DAPI. (Brightness settings untouched)……………………………14
Figure S13. Bioprinted MCF7 breast cancer cells stained with Neu, Phalloidin (green) and DAPI. (Brightness settings untouched)……………………………15
Figure S14. Bioprinted MCF7 breast cancer cells stained with Neu, Phalloidin (green) and DAPI. (Brightness settings slightly re-touched)………………..…16
Figure S15. Manually seeded MCF7 breast cancer cells stained with Neu, Phalloidin (green) and DAPI. (Brightness settings untouched)………………17
Figure S16. Manually seeded MCF7 breast cancer cells stained with Neu, Phalloidin (green) and DAPI. (Brightness settings untouched)………………18
Figure S17. Bioprinted MCF7 breast cancer cells stained with DAPI and PKH-67 (green fluorescence)…………………………………………………………..19
Figure S18. Manually seeded MCF7 breast cancer cells stained with DAPI and PKH-67 (green fluorescence)………………………………………………..20
Figure S19. Bioprinted MCF7 breast cancer cells 18 hours post bioprinting, not stained…………………………………………………………………………..21
Figure S20. Bioprinted MCF7 breast cancer cells 18 hours post bioprinting, not stained……………………………………………………………………..…22
Figure S21. Bioprinted MCF7 breast cancer cells 72 hours post bioprinting, not stained…………………………………………………………….............23
Figure S22. Manually seeded MCF7 breast cancer cells, prior to bioprinting, not stained…………………………………………………………………………24
Figure S23. Bioprinted MCF7 breast cancer cells 7 days post bioprinting, not stained (0.25X)………………………………………………………………………25
Figure S24. Manually seeded MCF7 breast cancer cells days post bioprinting, not stained 0.25x)………………...……………………………………………26
Figure S25. (A) Bioprinted and (B) Manually seeded MCF7 breast cancer cells………………………………………………………………………………27

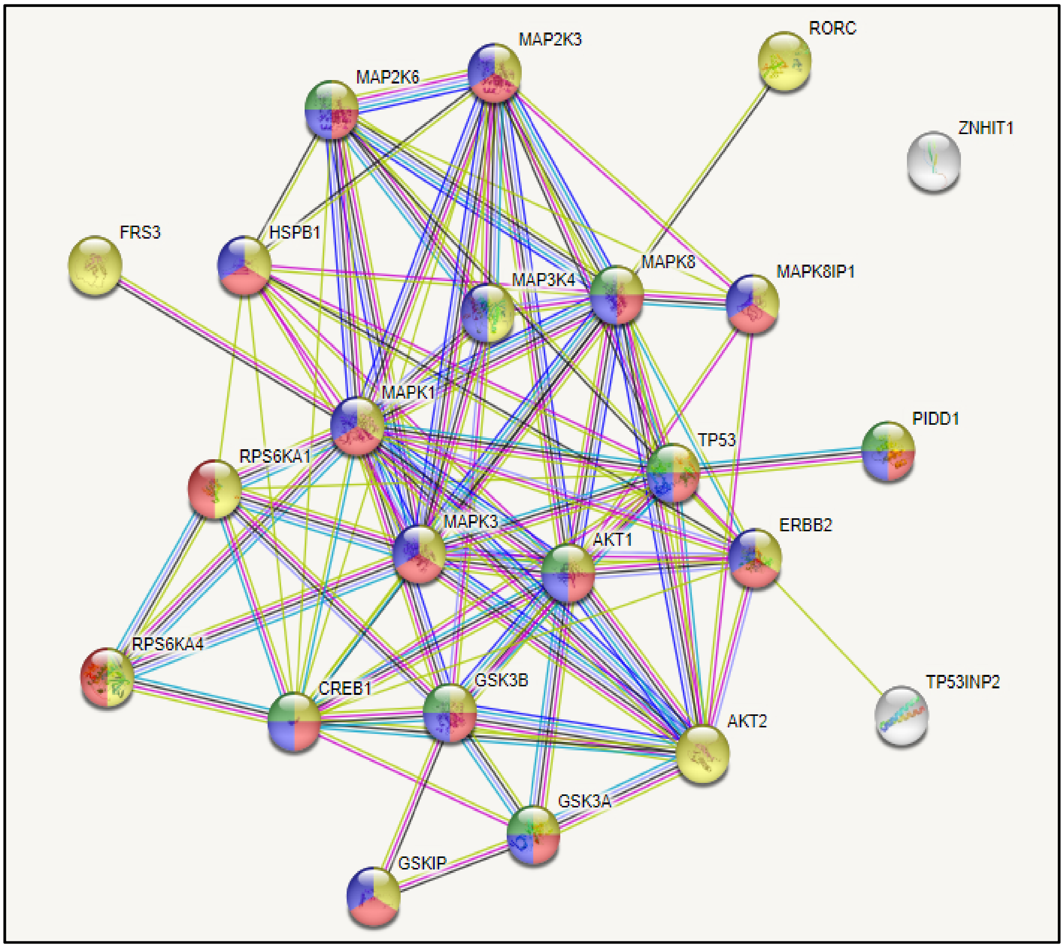

Figure S1. Network of analytes phosphorylated in BP MCF7 breast cancer cells. Functions selected in this network were regulator of apoptosis (green, 8), response to stress (red, 17), intracellular signal transduction (yellow, 21) and signal regulators (blue, 16). This network depicts functional interactions among BP BC predisposed genes. In this network of phosphorylated sites, there are significantly more interactions than expected (*p-value ≤ .001)*.

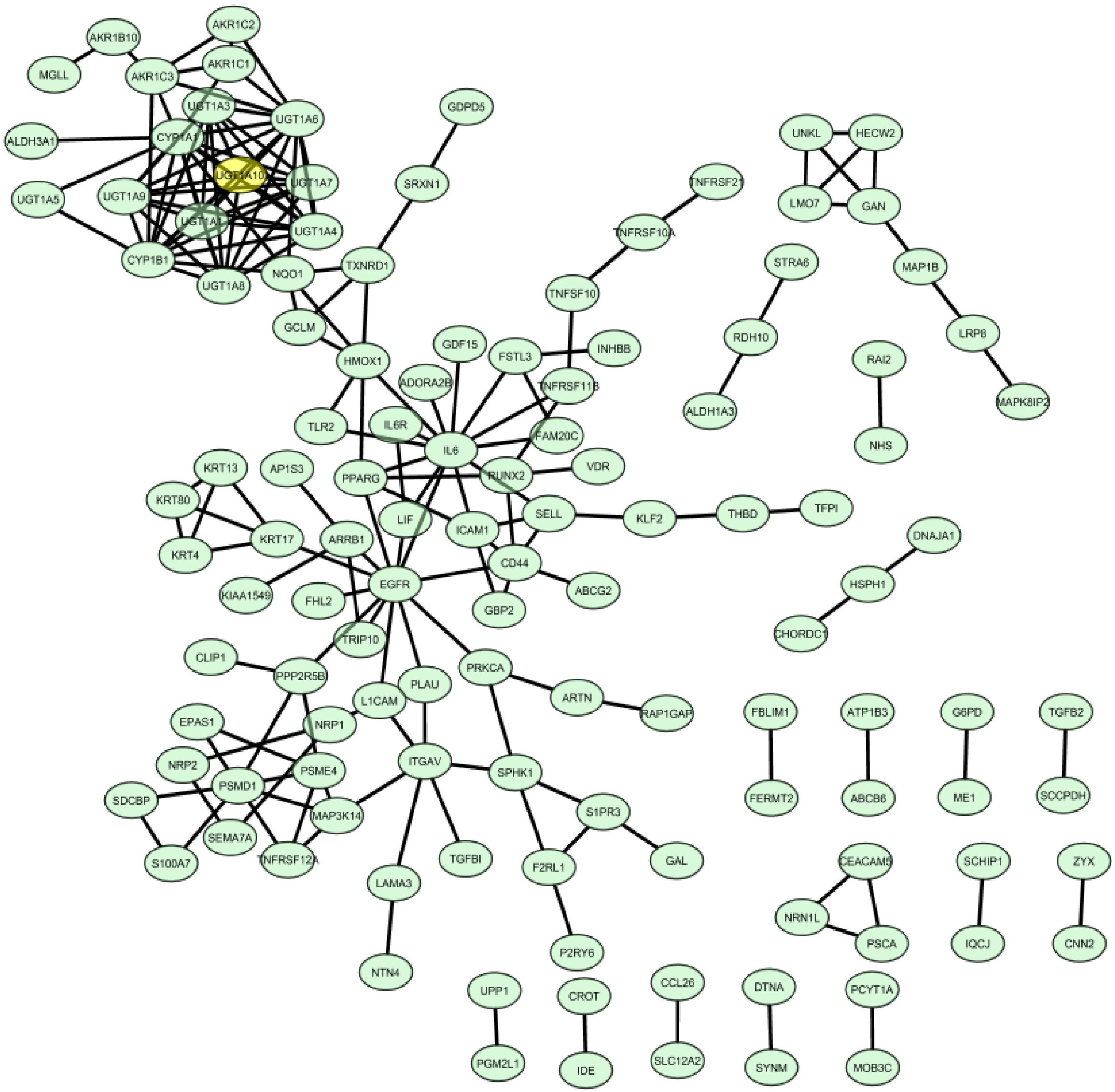

Figure S2. Network diagram of upregulated genes in RNA seq analysis, protein-protein network interaction, with organic layout, slightly re-arranged for better readability. Genes with >10 edges are observed for CYP1A, IL6, UGT1A6, EGFR and CYP1B1, (see *Table 1, from manuscript*). Ovals with pink fill were found to be involved in cell differentiation, ovals with yellow fill are involved in inflammatory response and ovals with green fill were identified as responding to stress.

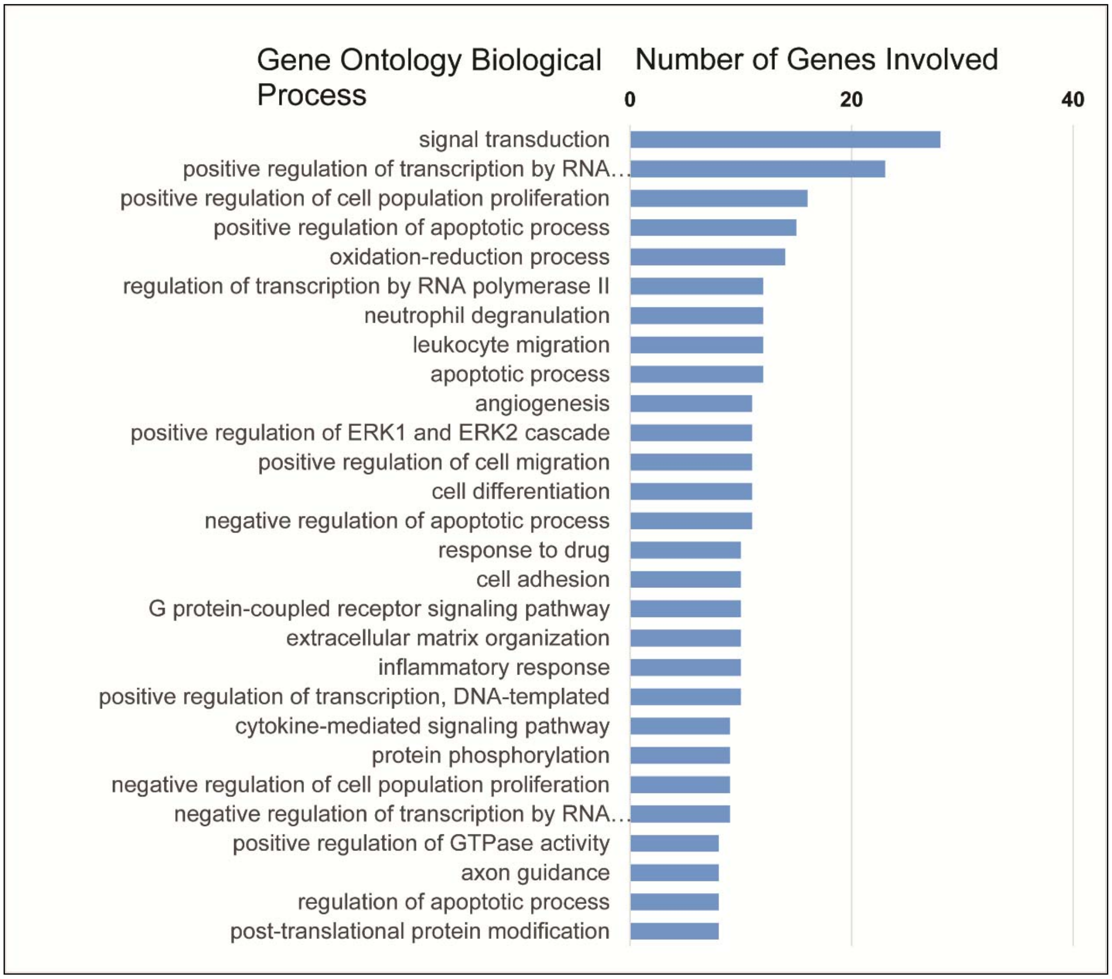

Figure S3. Bar graphs depicting frequency of the different molecular functions or sites associated with the GO terms in the differentially expressed genes (upregulated). The bars indicate the number of times a function or component is repeated, graph B displays the data connected to the biological processes. Data set is shown for > 8 repeated ontologies with *q-values* ≤ 0.05. The top three biological processes involved molecular signaling from the outside of the cell to the inside, inducing transcription and cell proliferation, which could be the cellular response to the stress these cells were exposed to.

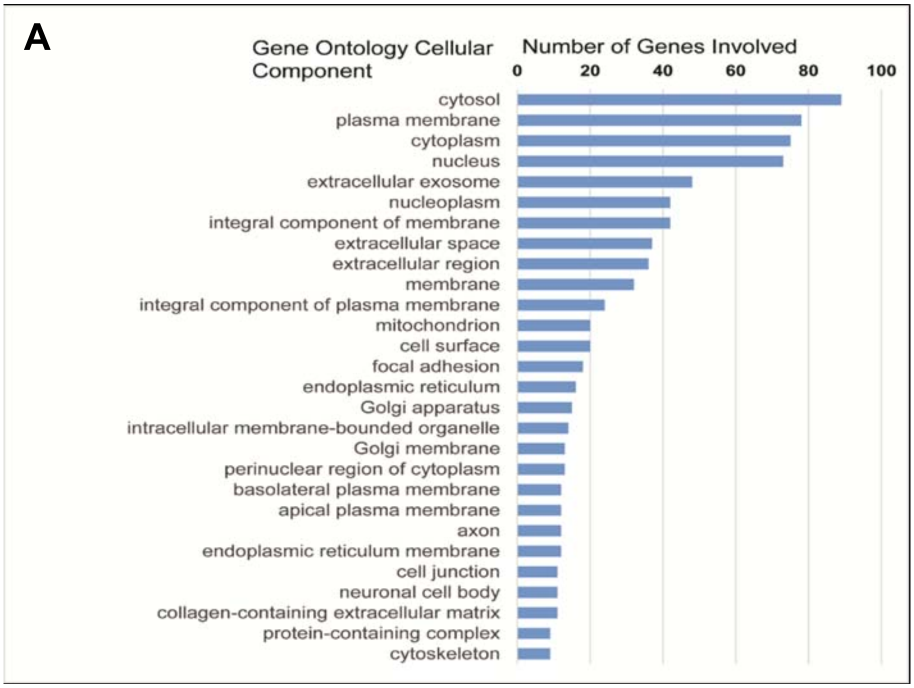

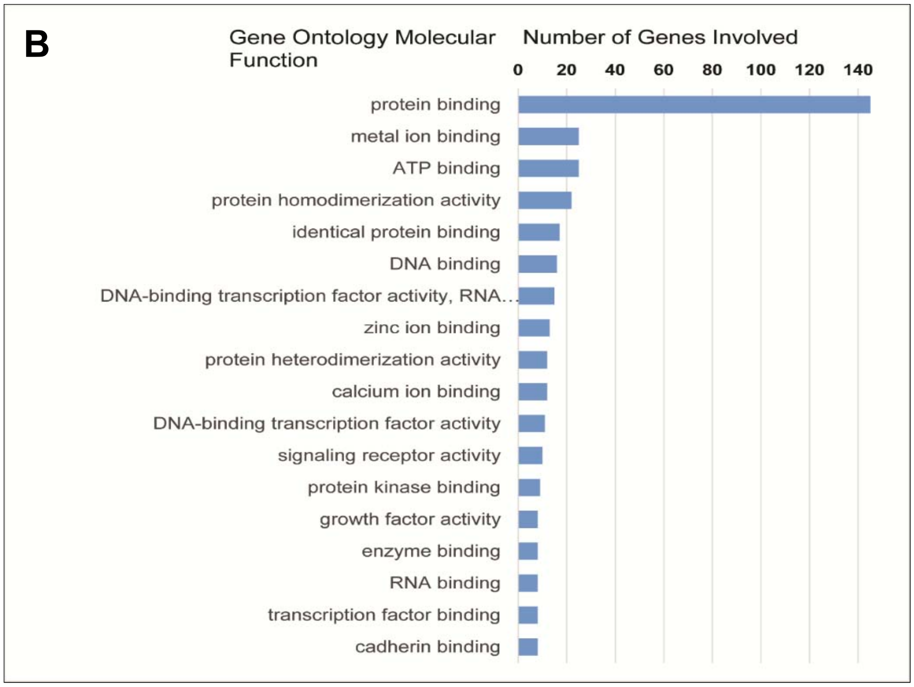

Figure S4. Bar graph displays data associated with the (A) molecular functions, and (B) cellular components. Data set is shown for > 8 repeated ontologies with *q-values* ≤ 0.05. The top three biological processes involved molecular signaling from the outside of the cell to the inside, inducing transcription and cell proliferation, which could be the cellular response to the stress these cells were exposed to.

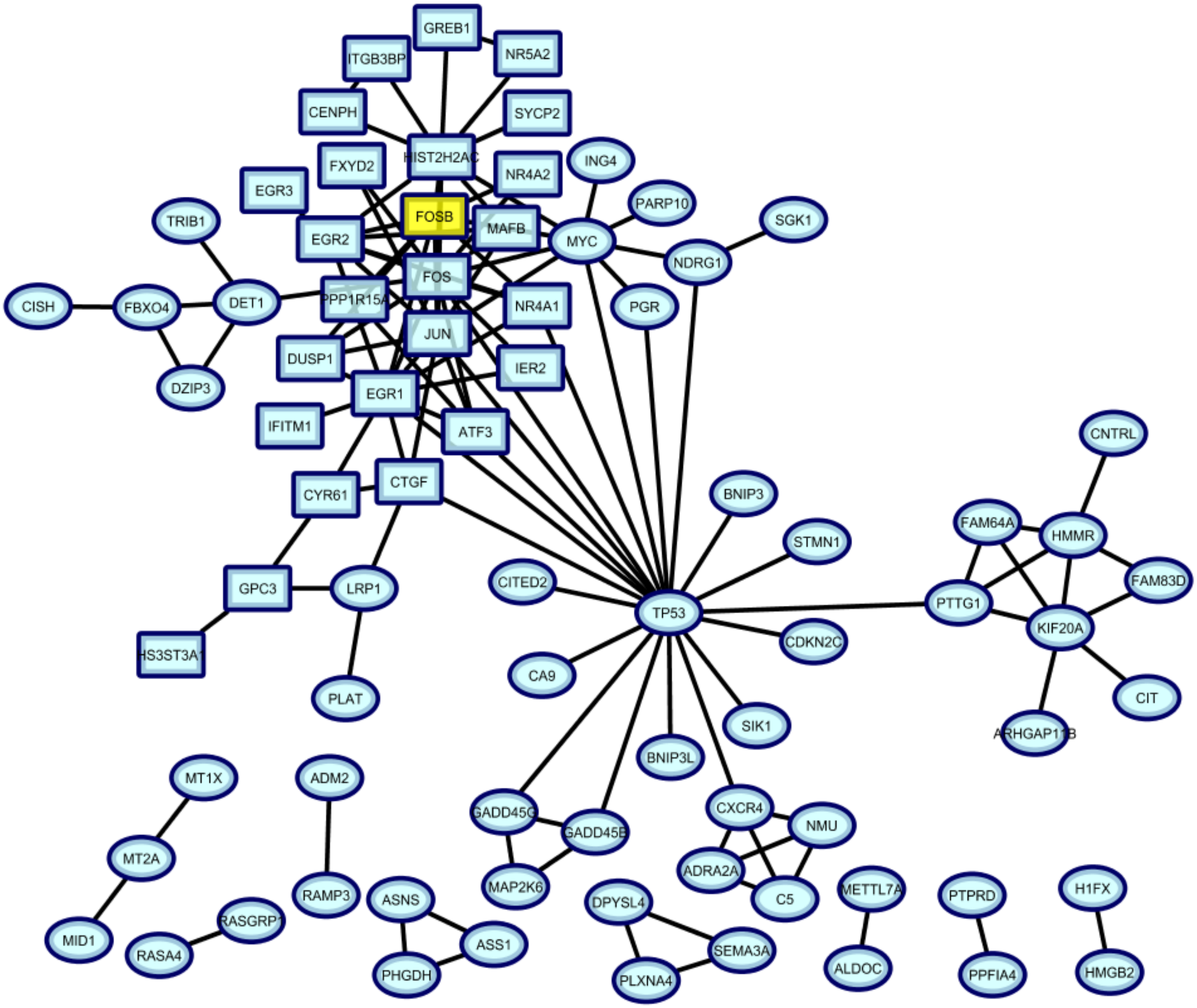

Figure S5. Network diagram of downregulated genes in RNA seq analysis of BP MCF7 BCCs, protein-protein network interaction, layout selected was organic layout, slightly re-arranged for better readability. This network contains only the genes defined in STRING. Genes with >10 edges are observed for TP53, FOS, JUN, EGR1, HIST2, H2AC, and FOSB, (see *Table 2, in the manuscript*).

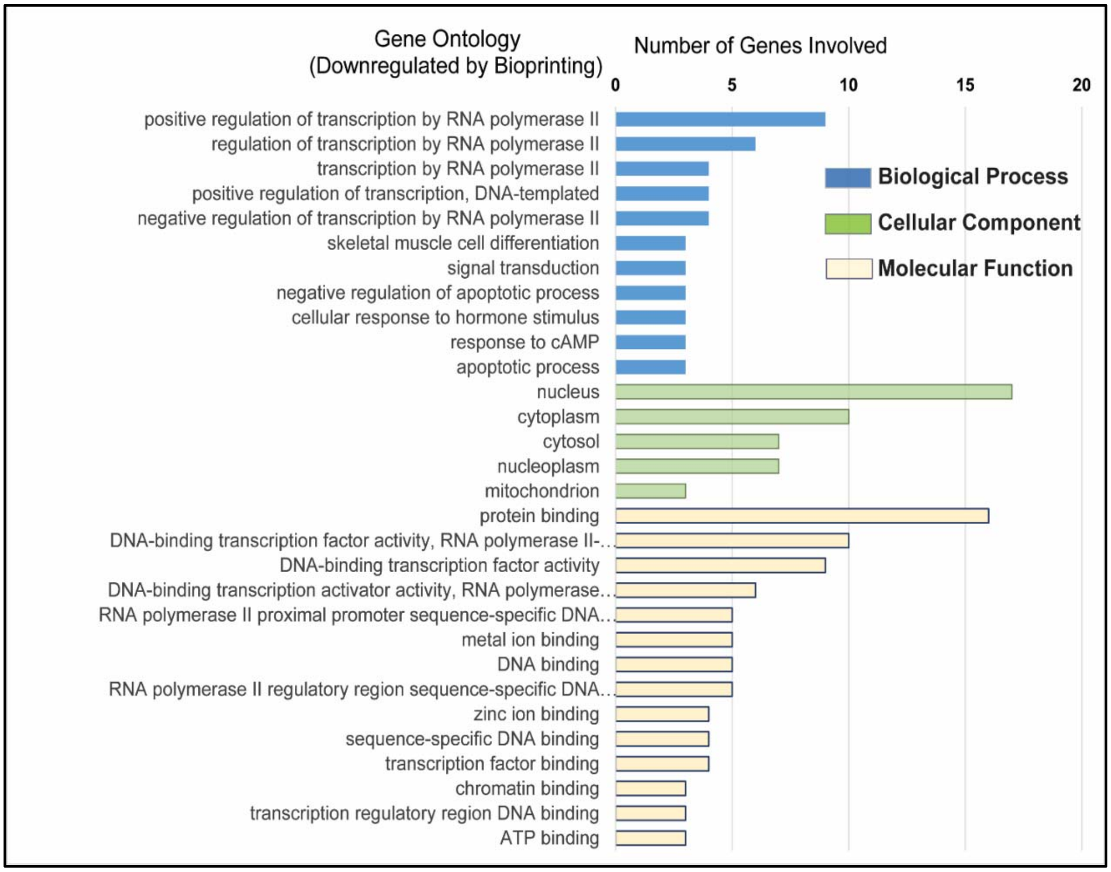

Figure S6. Bar graphs depicting frequency of the different molecular functions or sites associated with the GO terms in the differentially expressed genes (downregulated genes). Fewer ontologies under biological process were observed in downregulated genes due to bioprinting, which is what we expected to find. The bars indicate the number of times a function or component is repeated, data connected to the biological processes, molecular functions, and cellular components. Data set is shown for > 3 repeated ontologies with *q-values* ≤ 0.05.

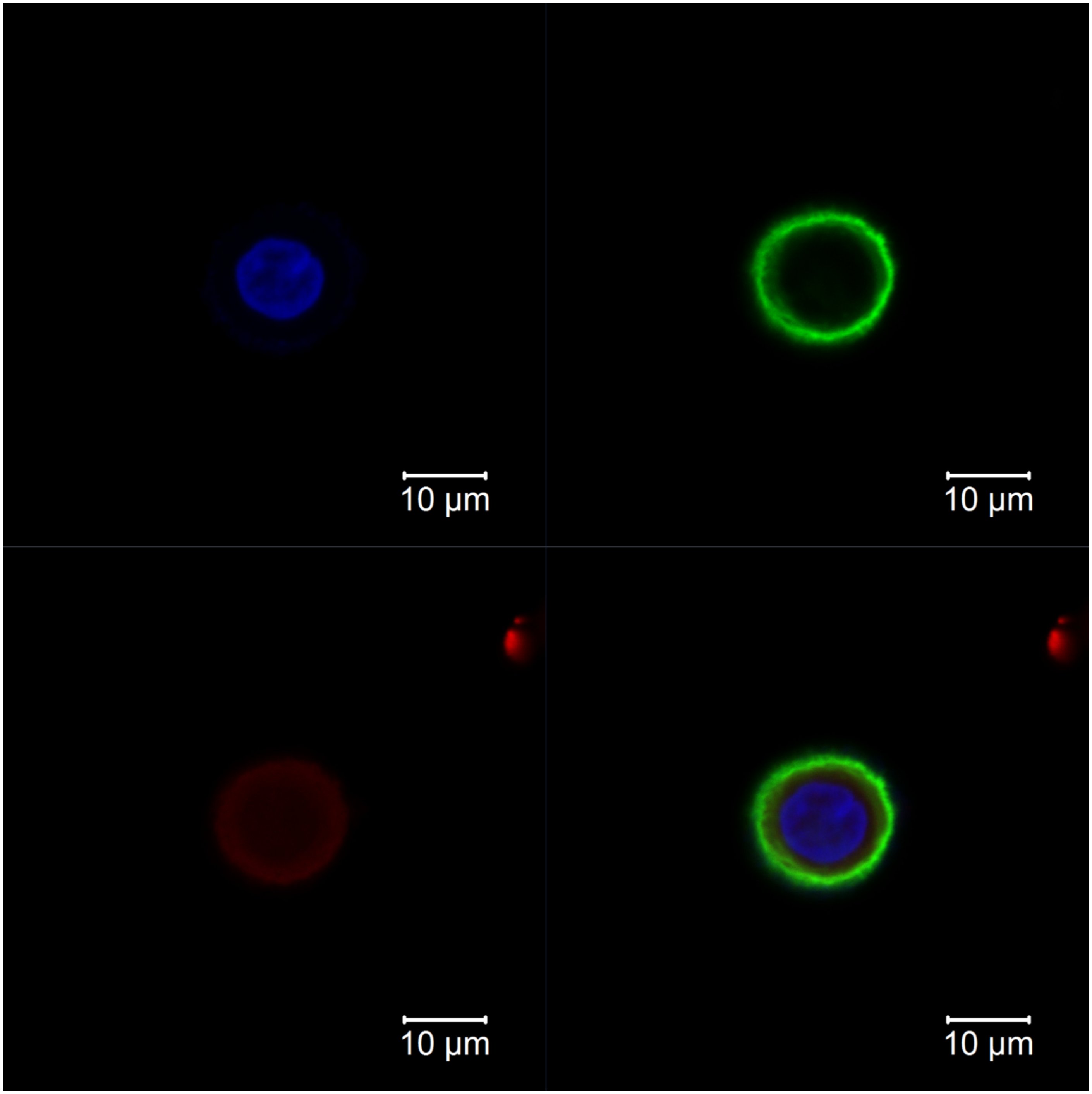

Figure S7. Bioprinted MCF7 breast cancer cells stained with Neu, Phalloidin (green) and DAPI. (Brightness settings untouched)

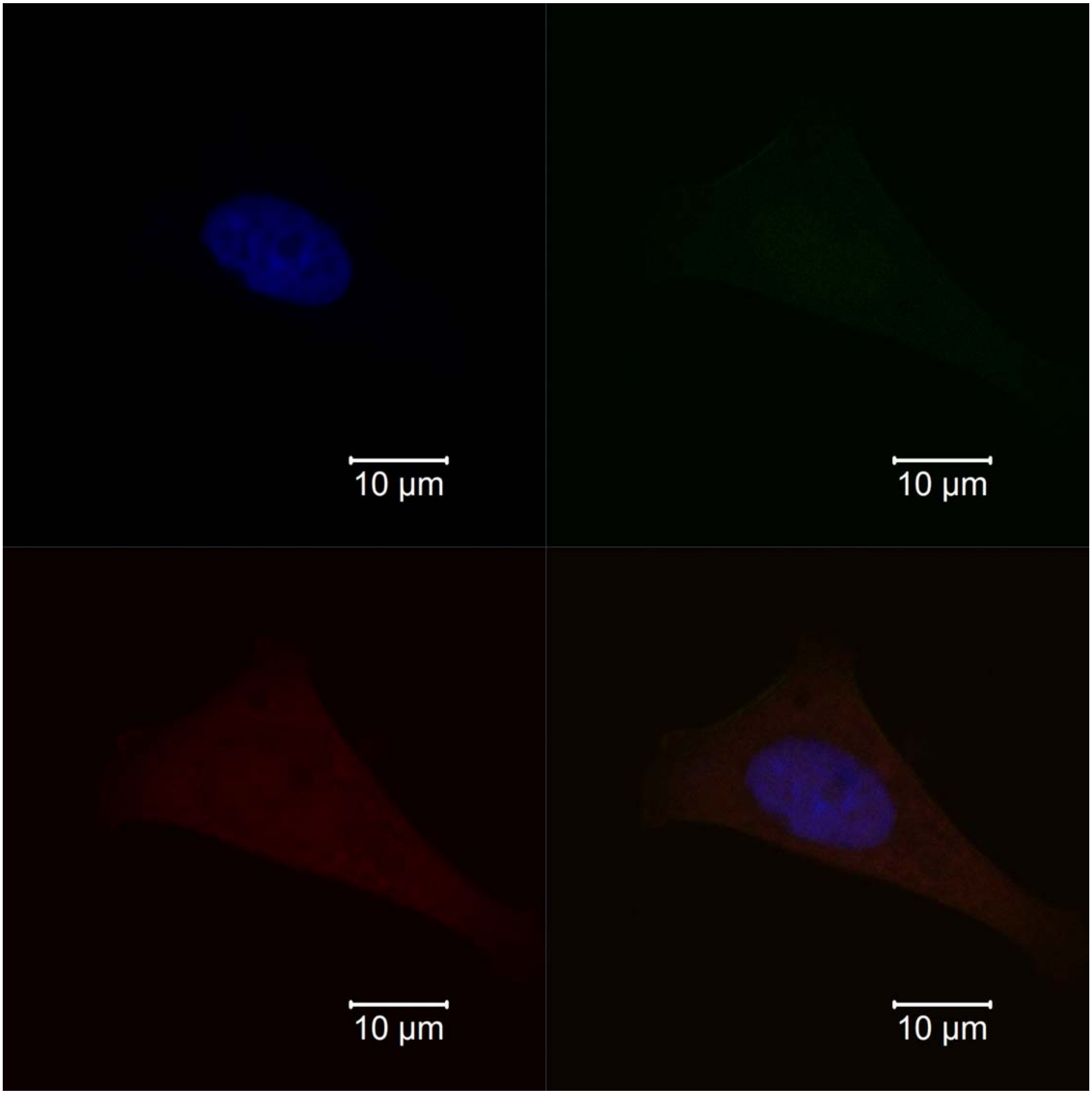

Figure S8. Bioprinted MCF7 breast cancer cells stained with Neu, Phalloidin (green) and DAPI. (Brightness settings untouched)

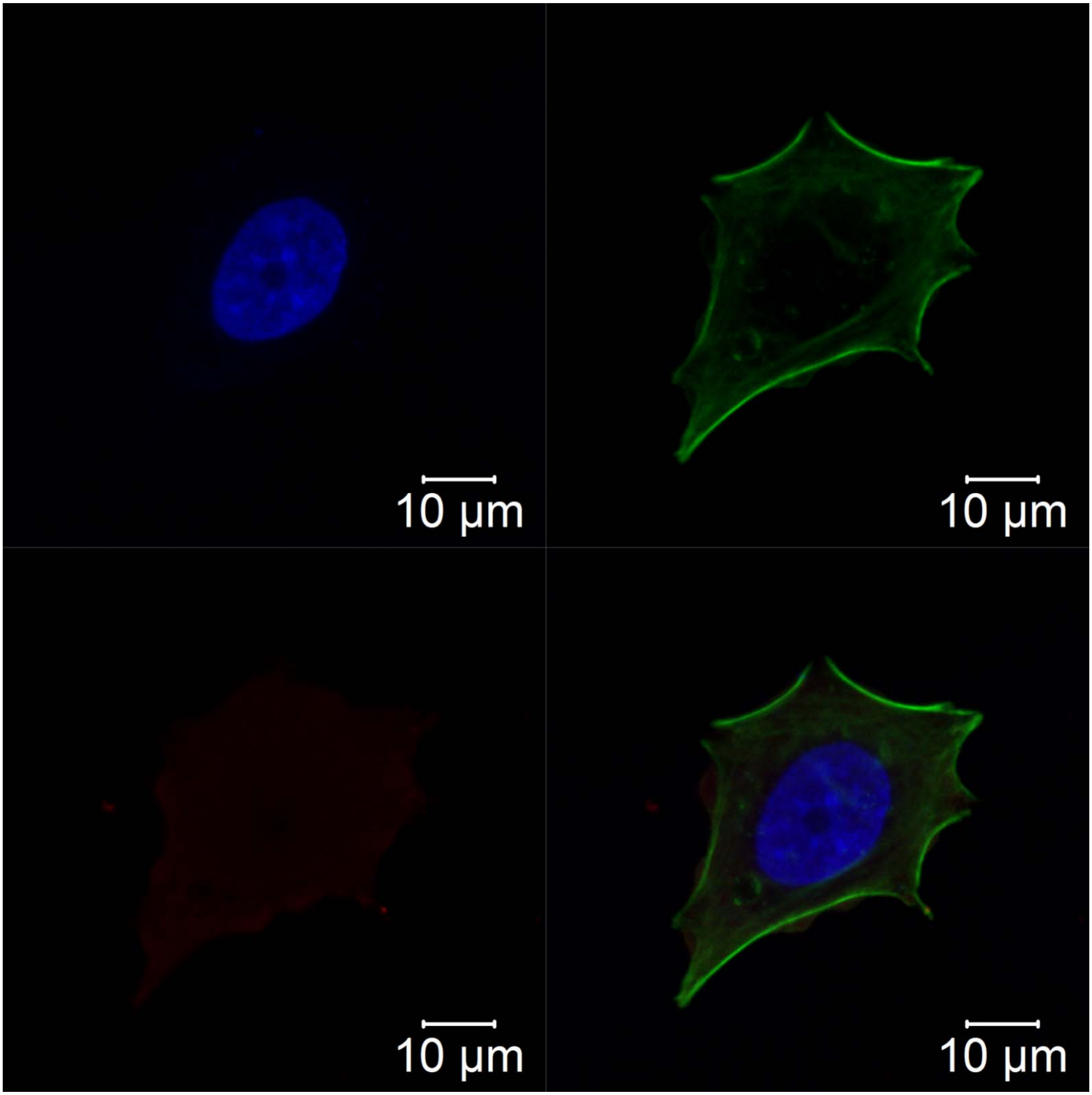

Figure S9. Bioprinted MCF7 breast cancer cells stained with Neu, Phalloidin (green) and DAPI. (Brightness settings untouched)

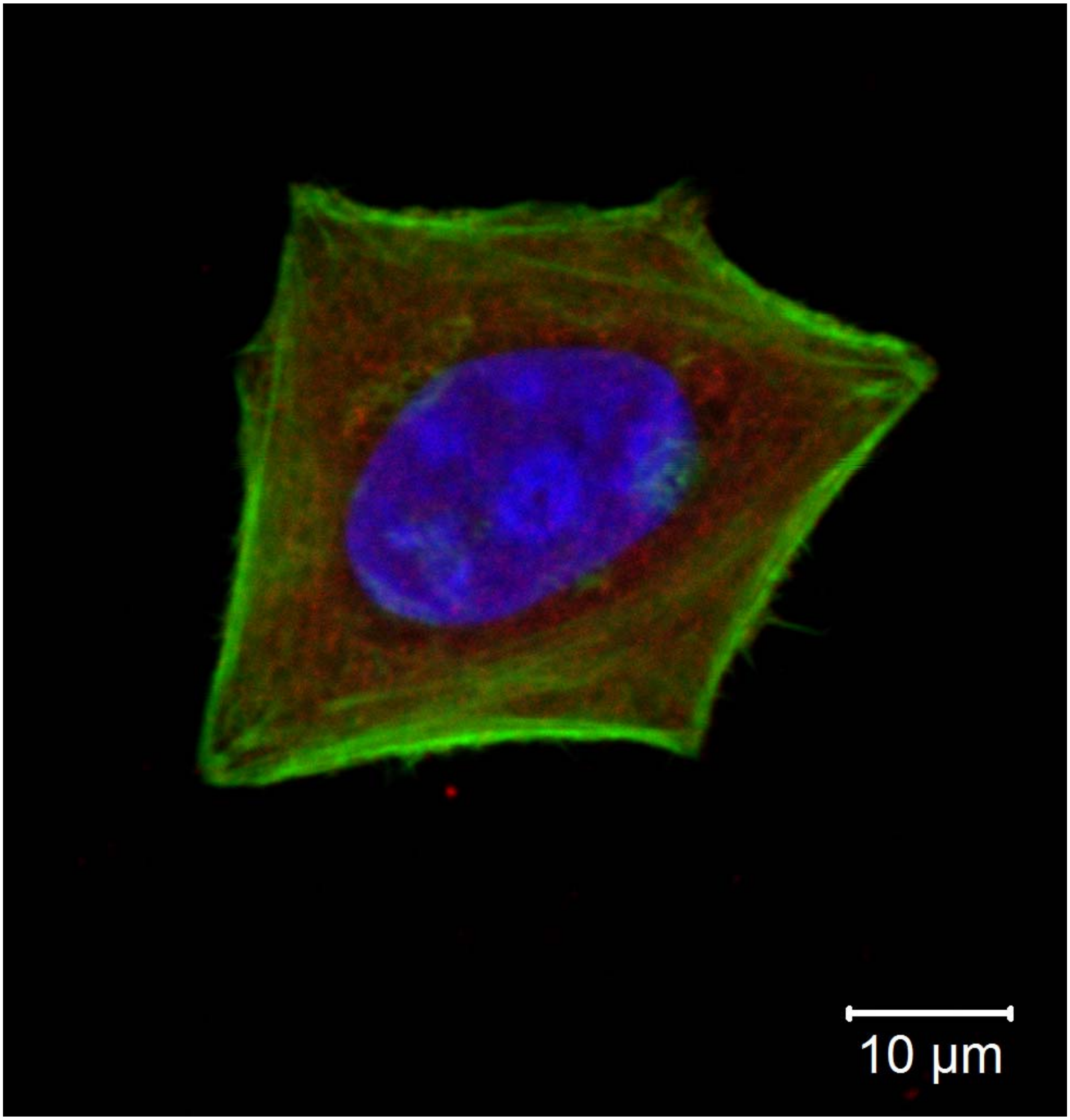

Figure S10. Bioprinted MCF7 breast cancer cells stained with Neu, Phalloidin (green) and DAPI. (Brightness settings slightly retouched)

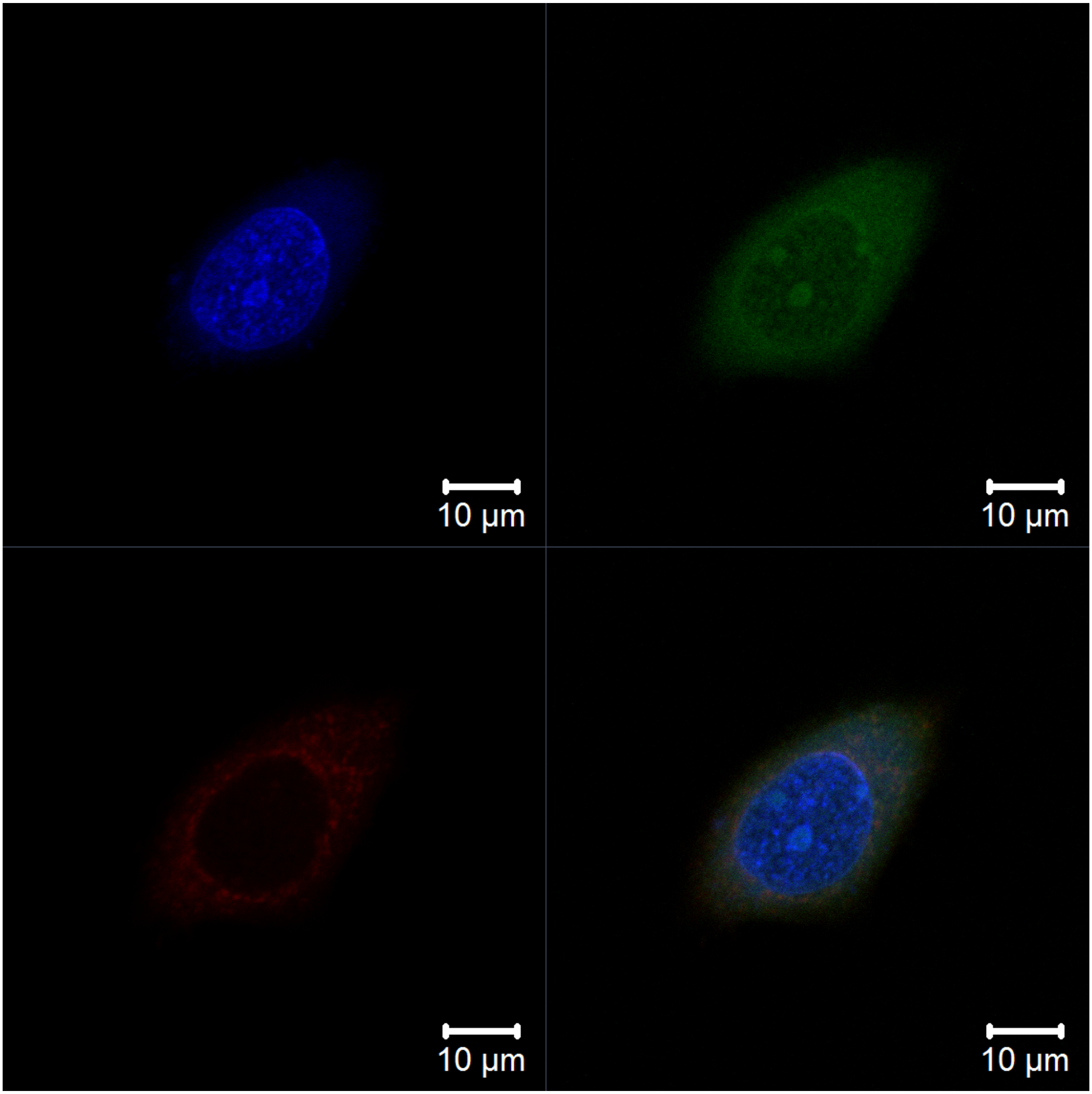

Figure S11. Bioprinted MCF7 breast cancer cells stained with Neu, Phalloidin (green) and DAPI. (Brightness settings untouched)

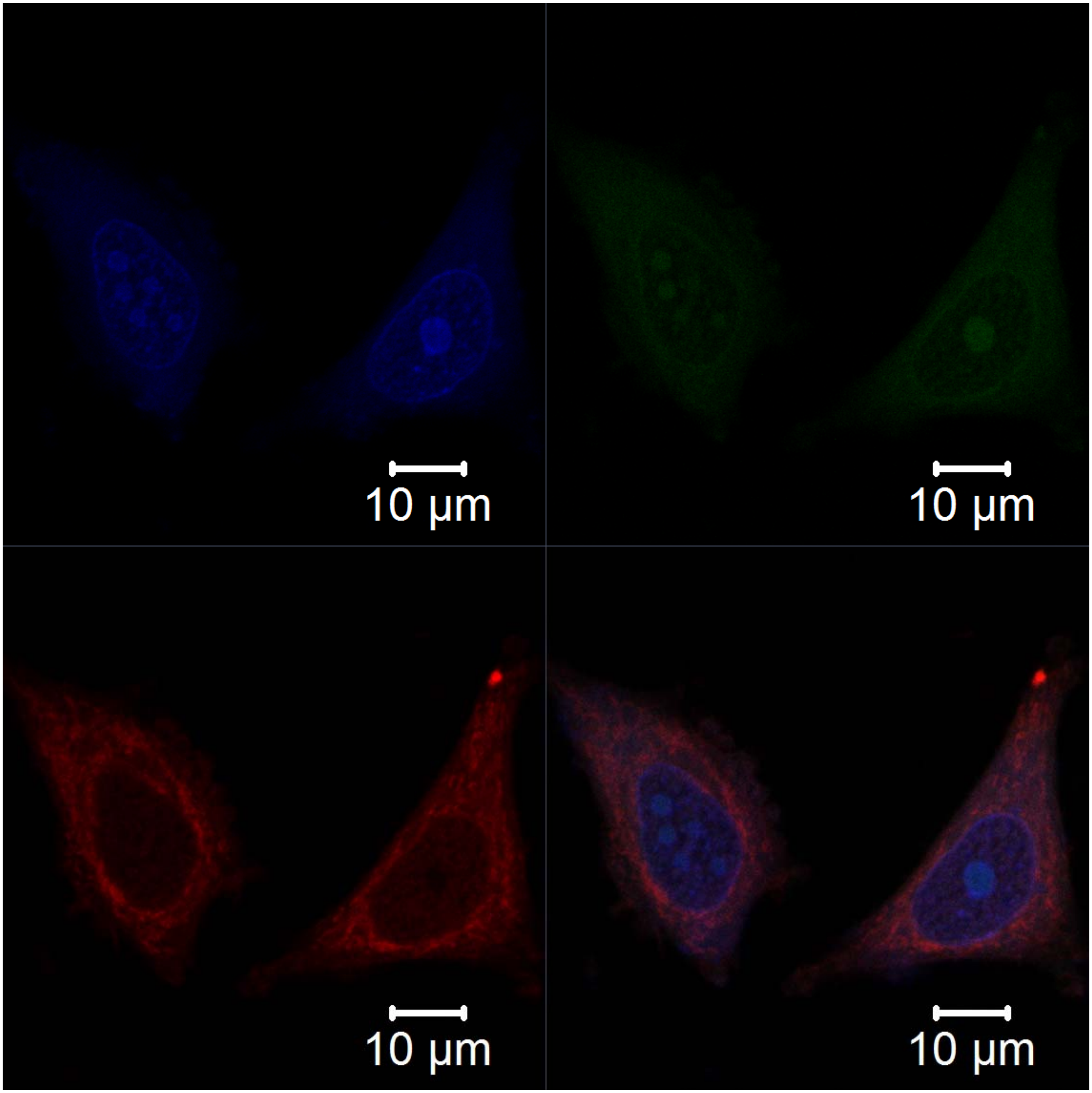

Figure S12. Bioprinted MCF7 breast cancer cells stained with Neu, Phalloidin (green) and DAPI. (Brightness settings untouched)

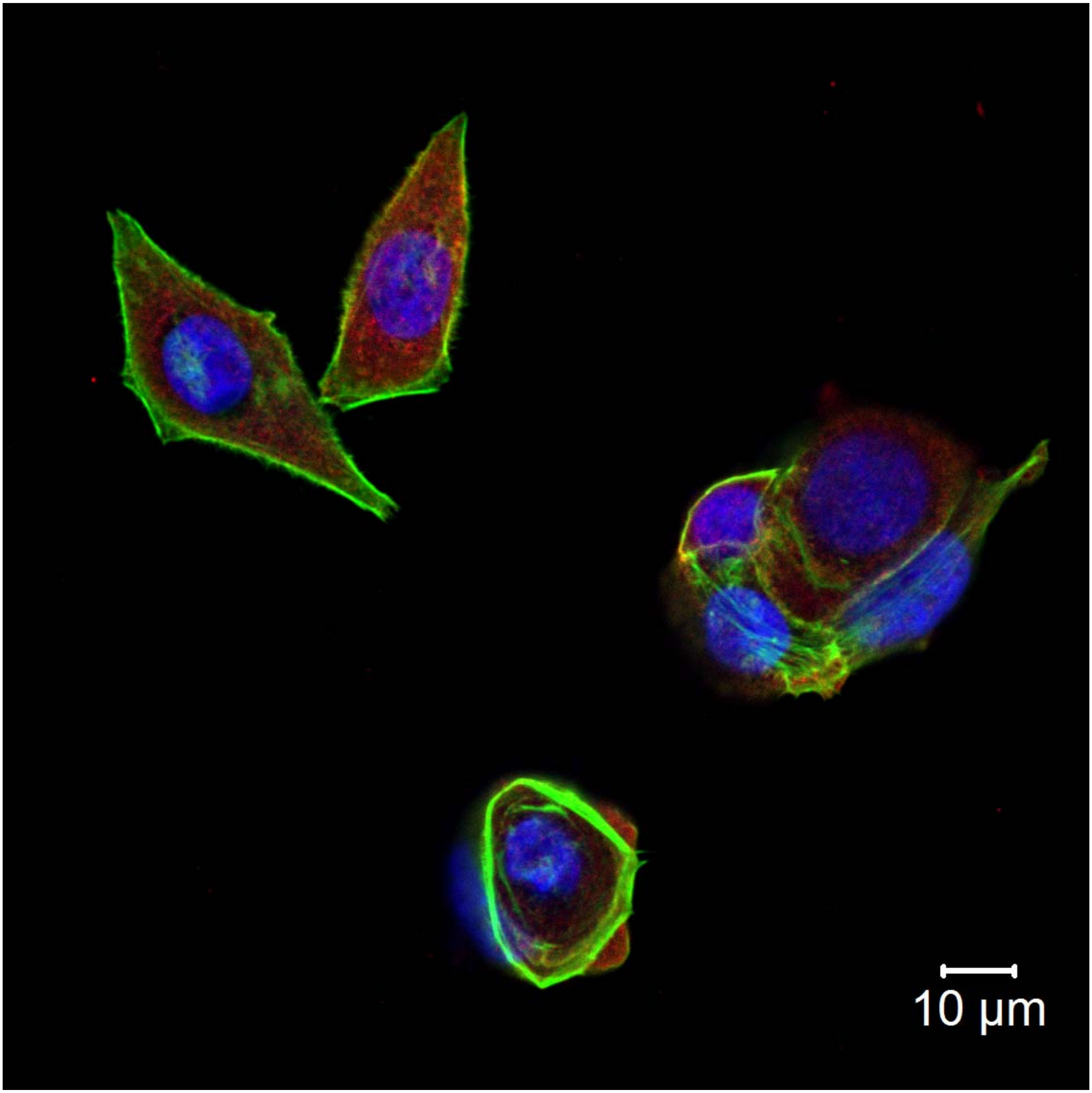

Figure S13. Bioprinted MCF7 breast cancer cells stained with Neu, Phalloidin (green) and DAPI. (Brightness settings untouched)

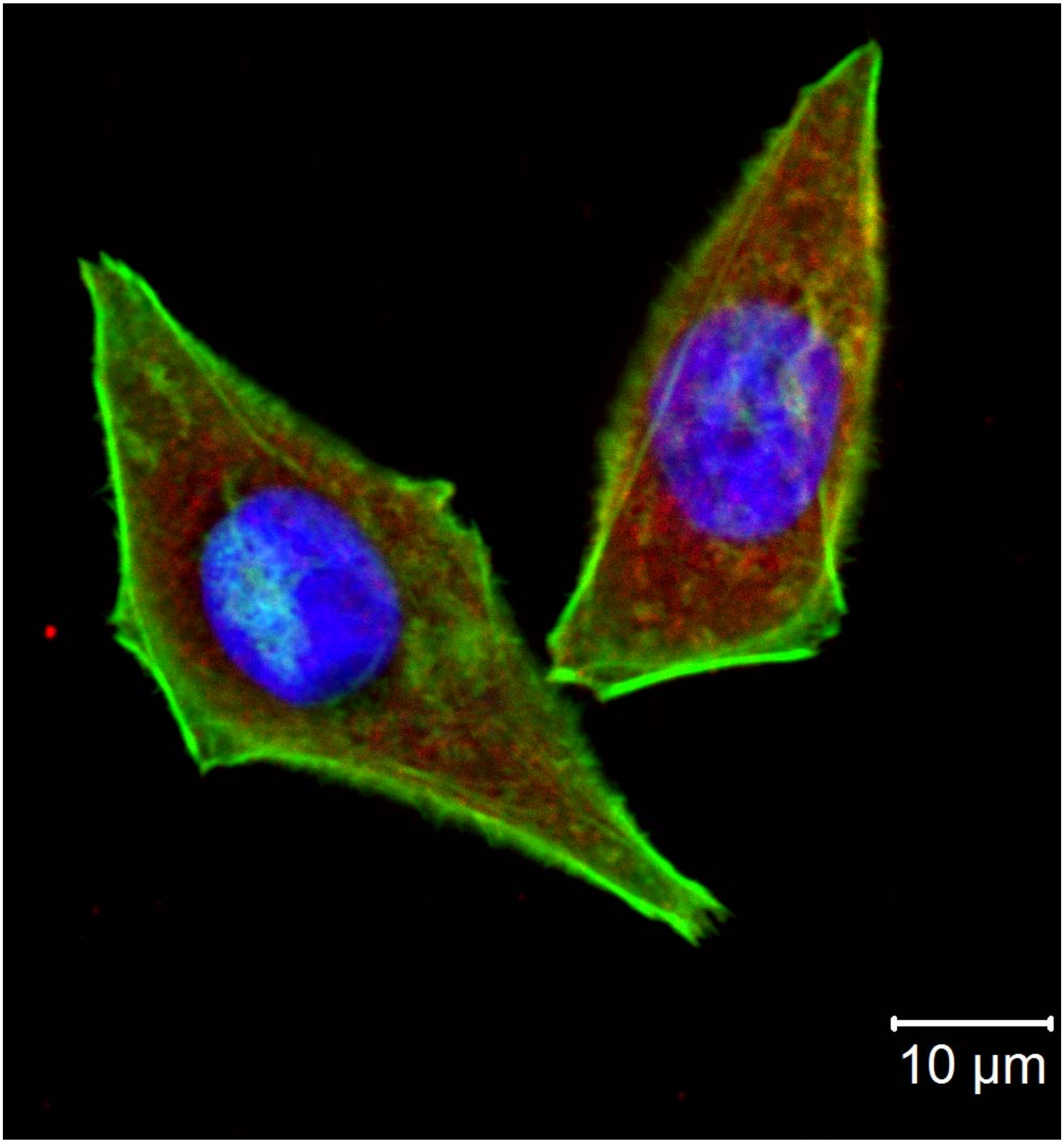

Figure S14. Bioprinted MCF7 breast cancer cells stained with Neu, Phalloidin (green) and DAPI. (Brightness settings slightly re-touched)

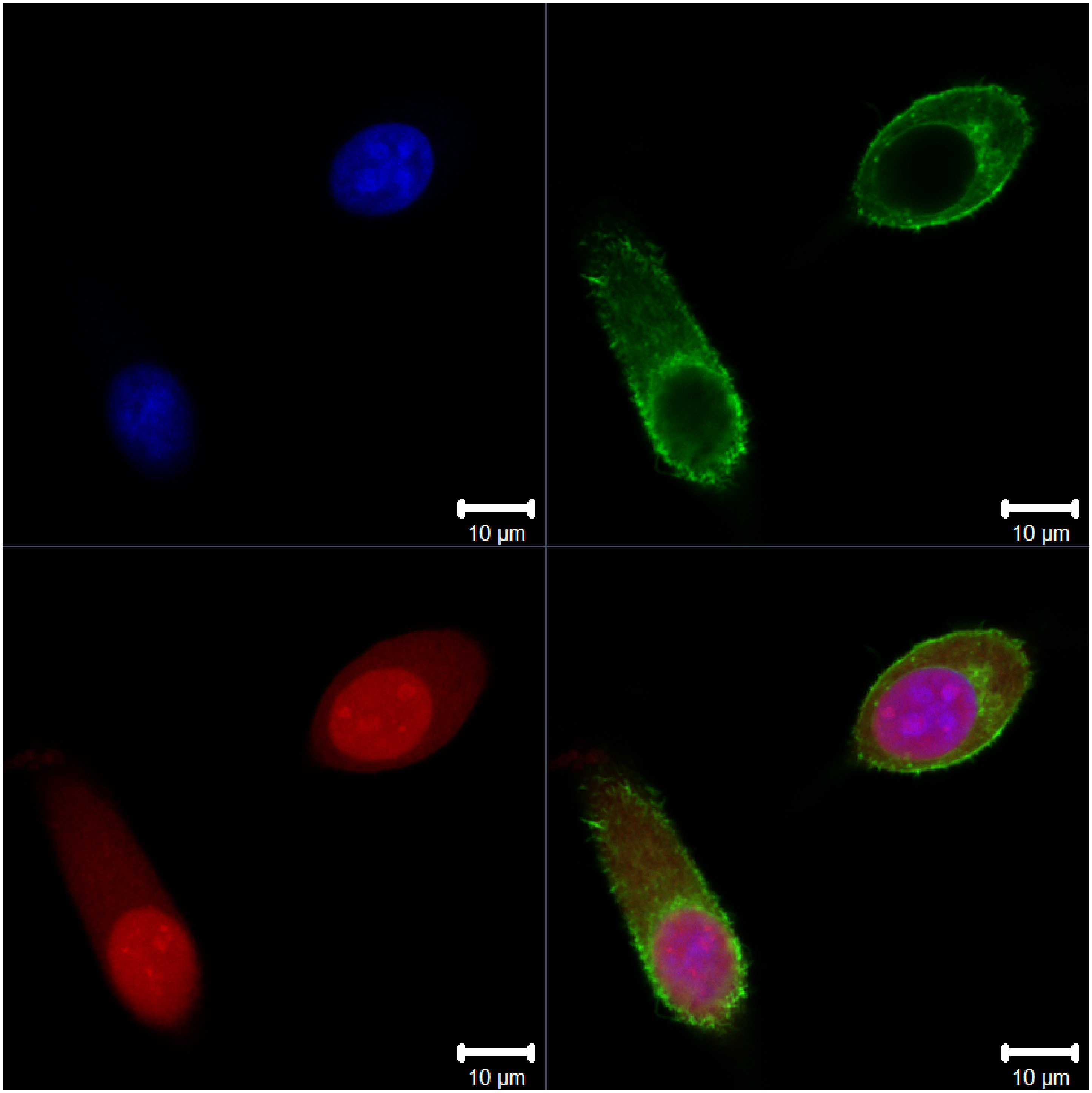

Figure S15. Manually seeded MCF7 breast cancer cells stained with Neu, Phalloidin (green) and DAPI. (Brightness settings untouched)

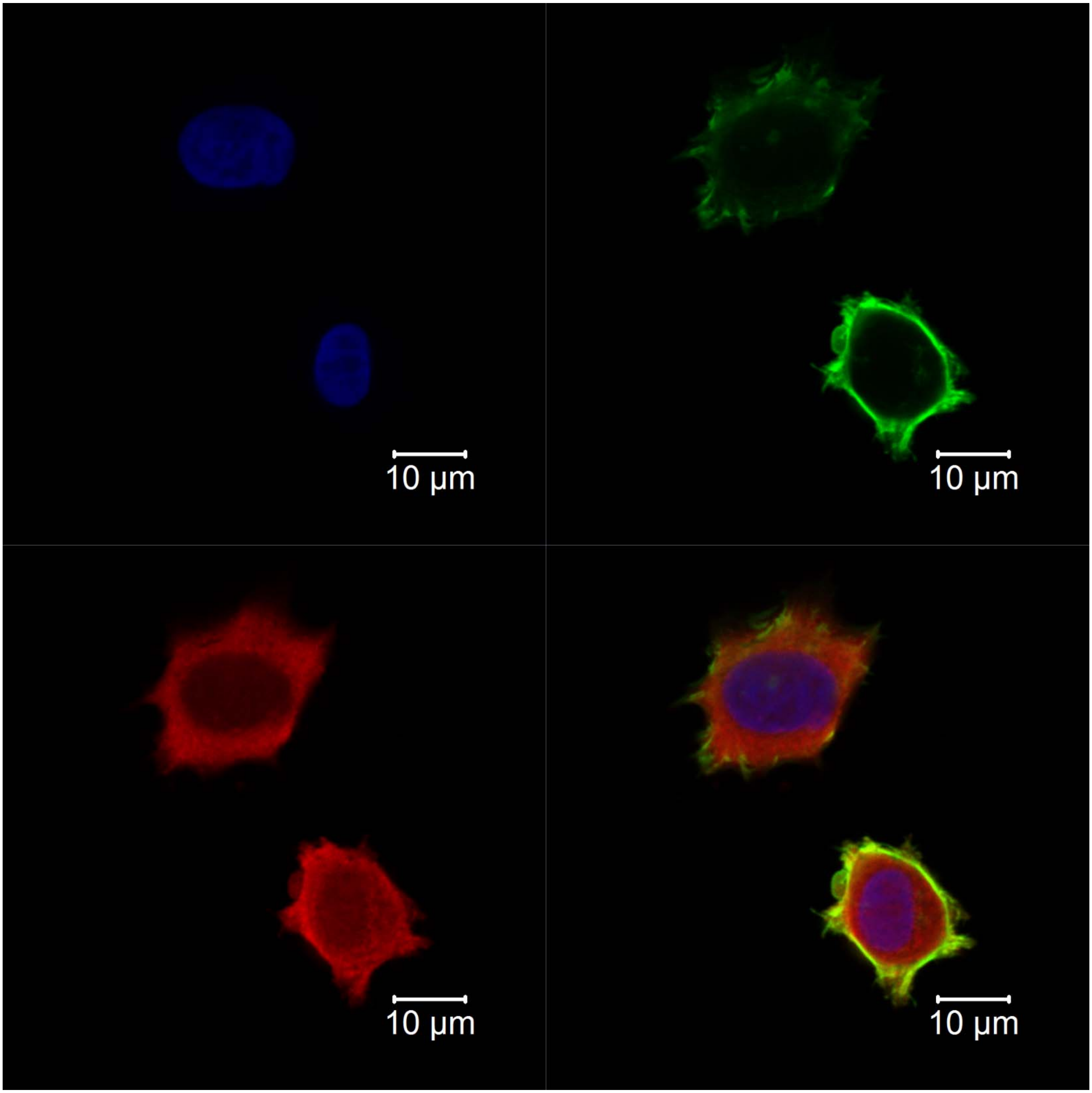

Figure S16. Manually seeded MCF7 breast cancer cells stained with Neu, Phalloidin (green) and DAPI. (Brightness settings untouched)

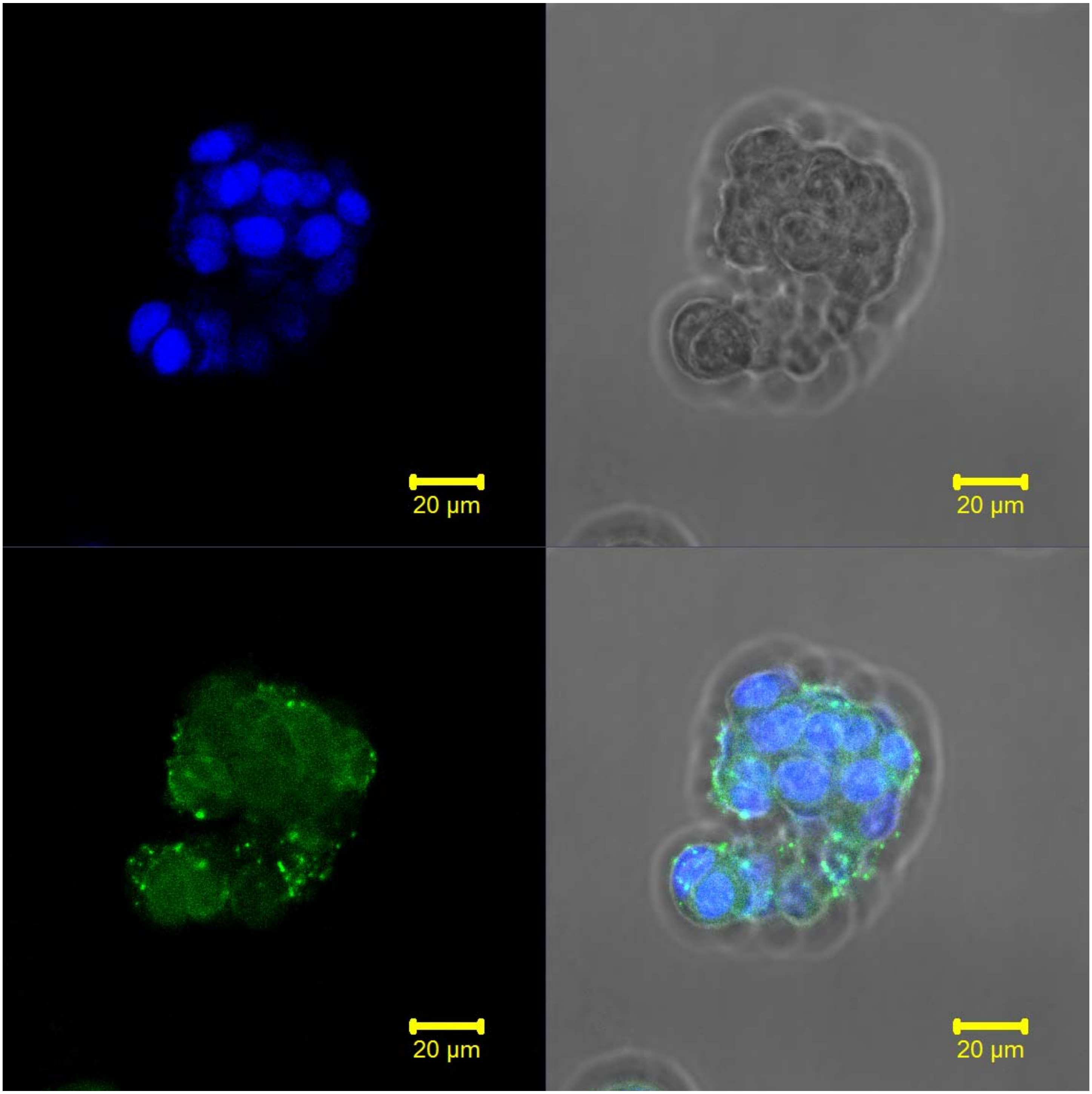

Figure S17. Bioprinted MCF7 breast cancer cells stained with DAPI and PKH-67 (green fluorescence).

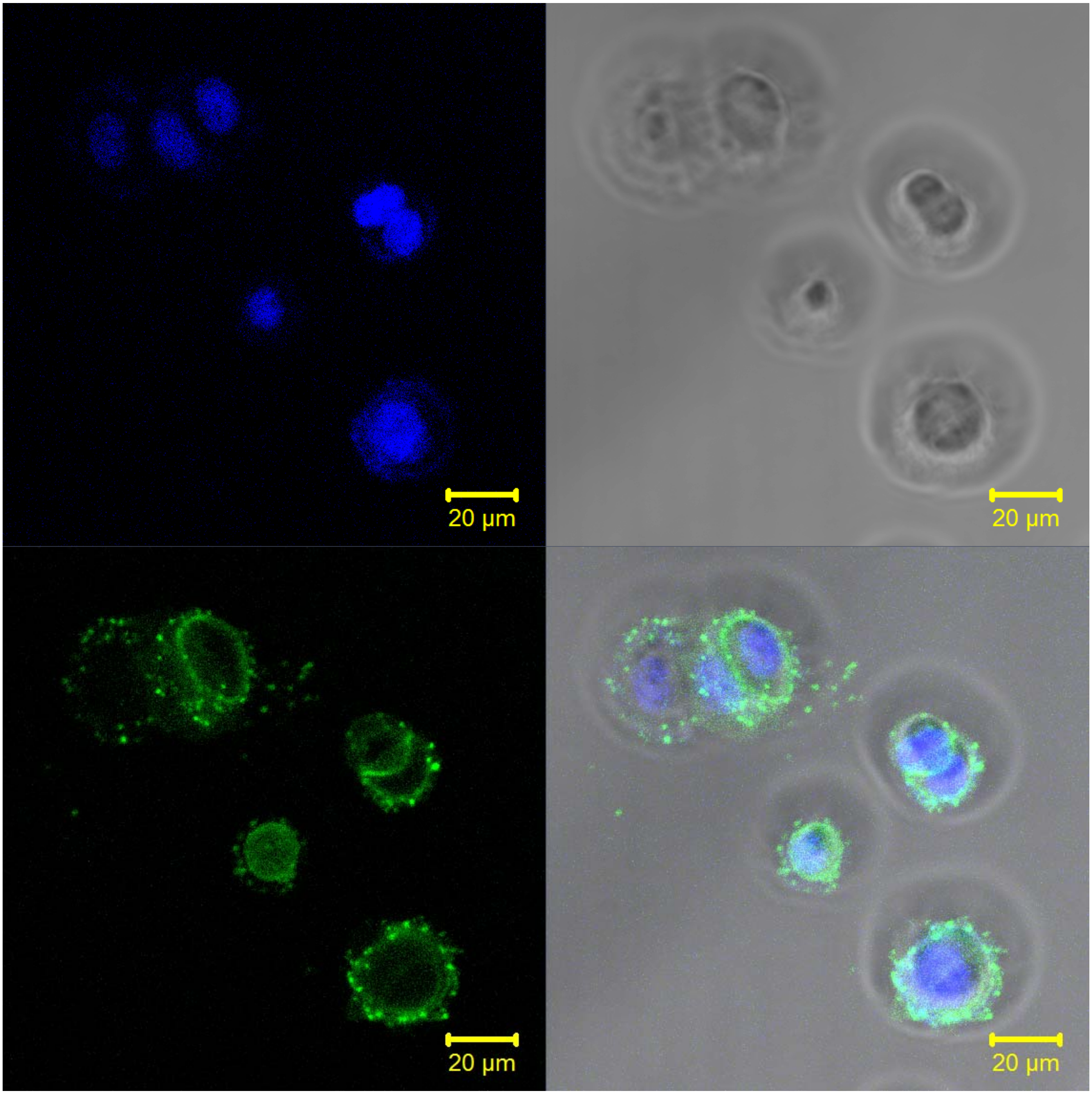

Figure S18. Manually seeded MCF7 breast cancer cells stained with DAPI and PKH-67 (green fluorescence).

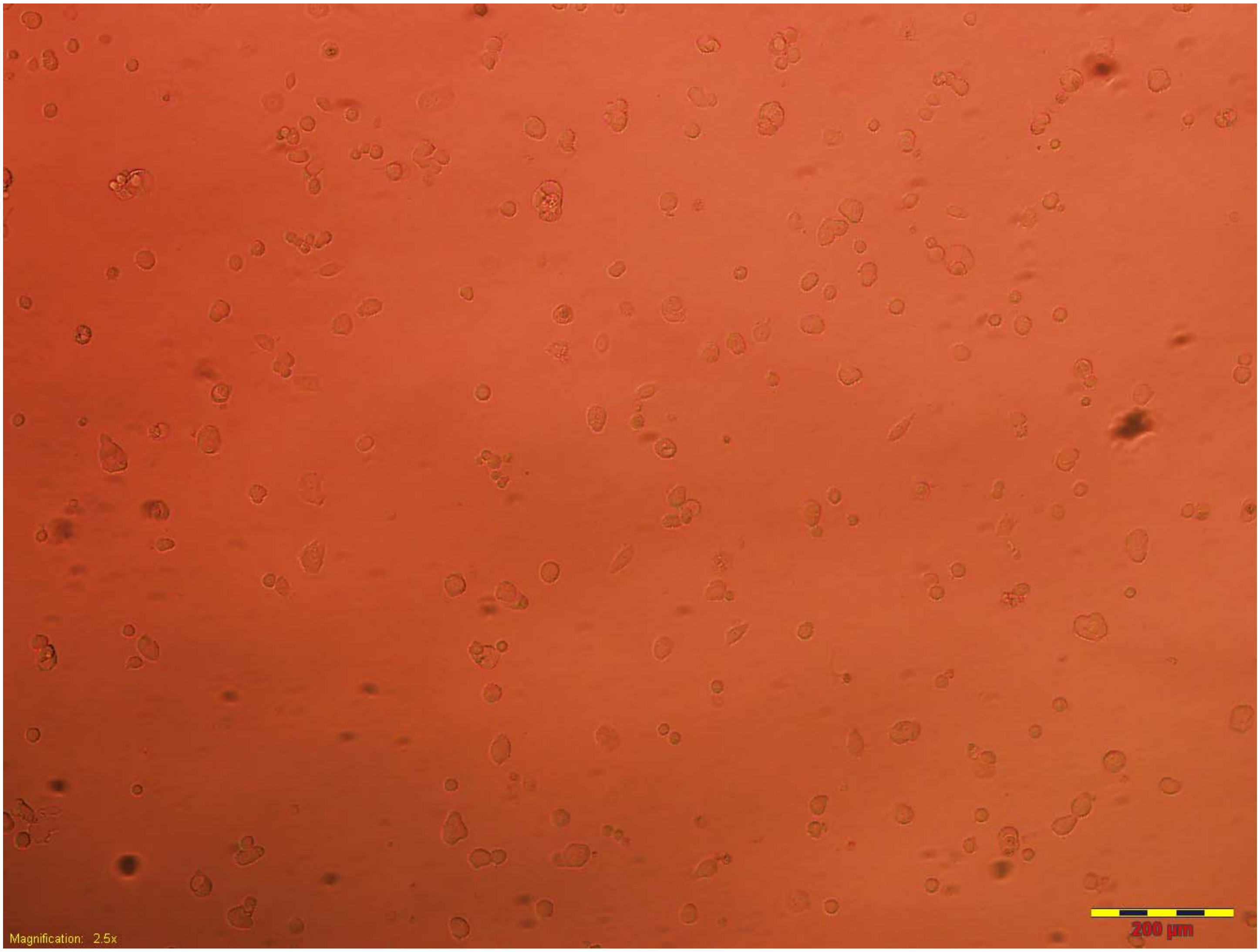

Figure S19. Bioprinted MCF7 breast cancer cells 18 hours post bioprinting, not stained

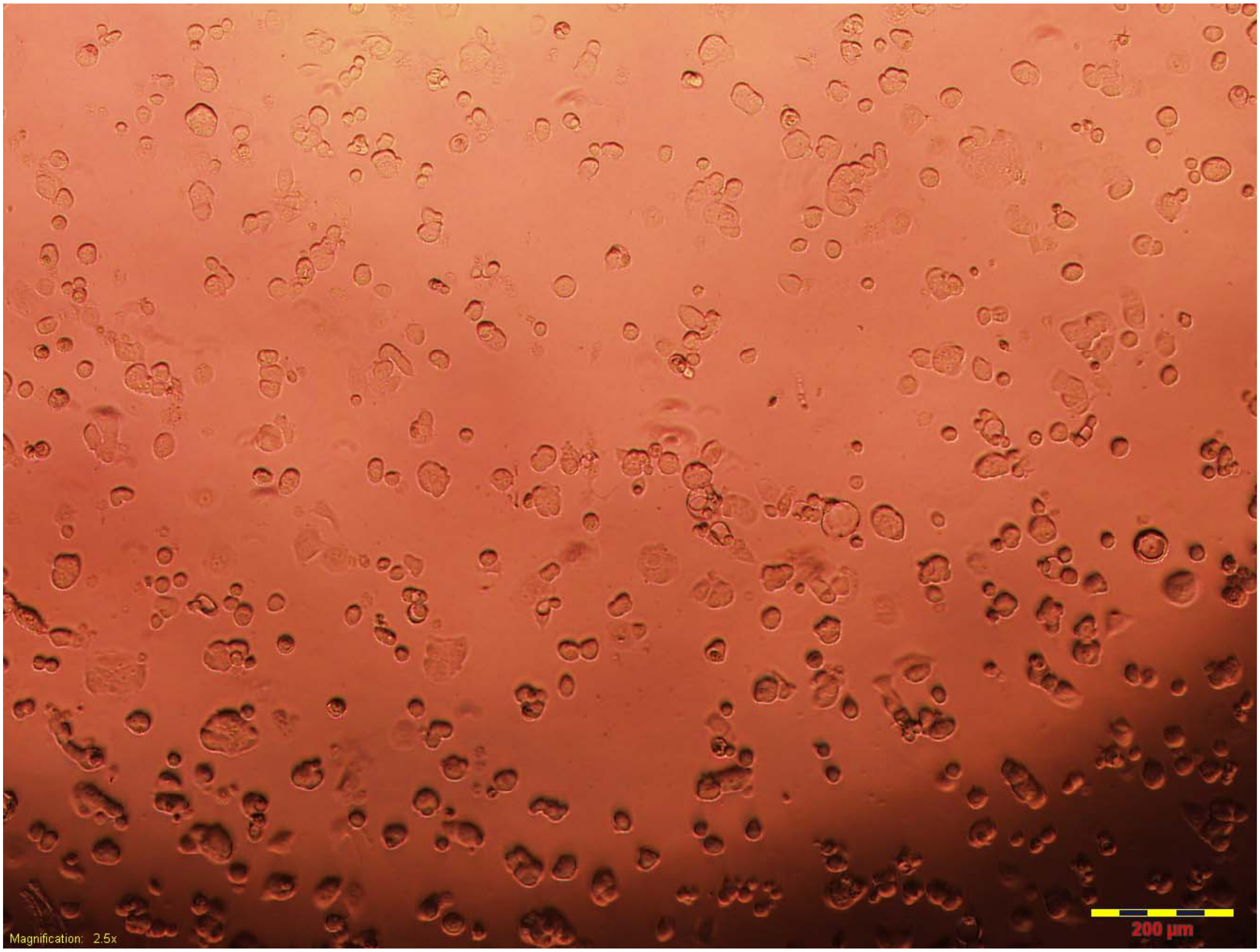

Figure S20. Bioprinted MCF7 breast cancer cells 18 hours post bioprinting, not stained

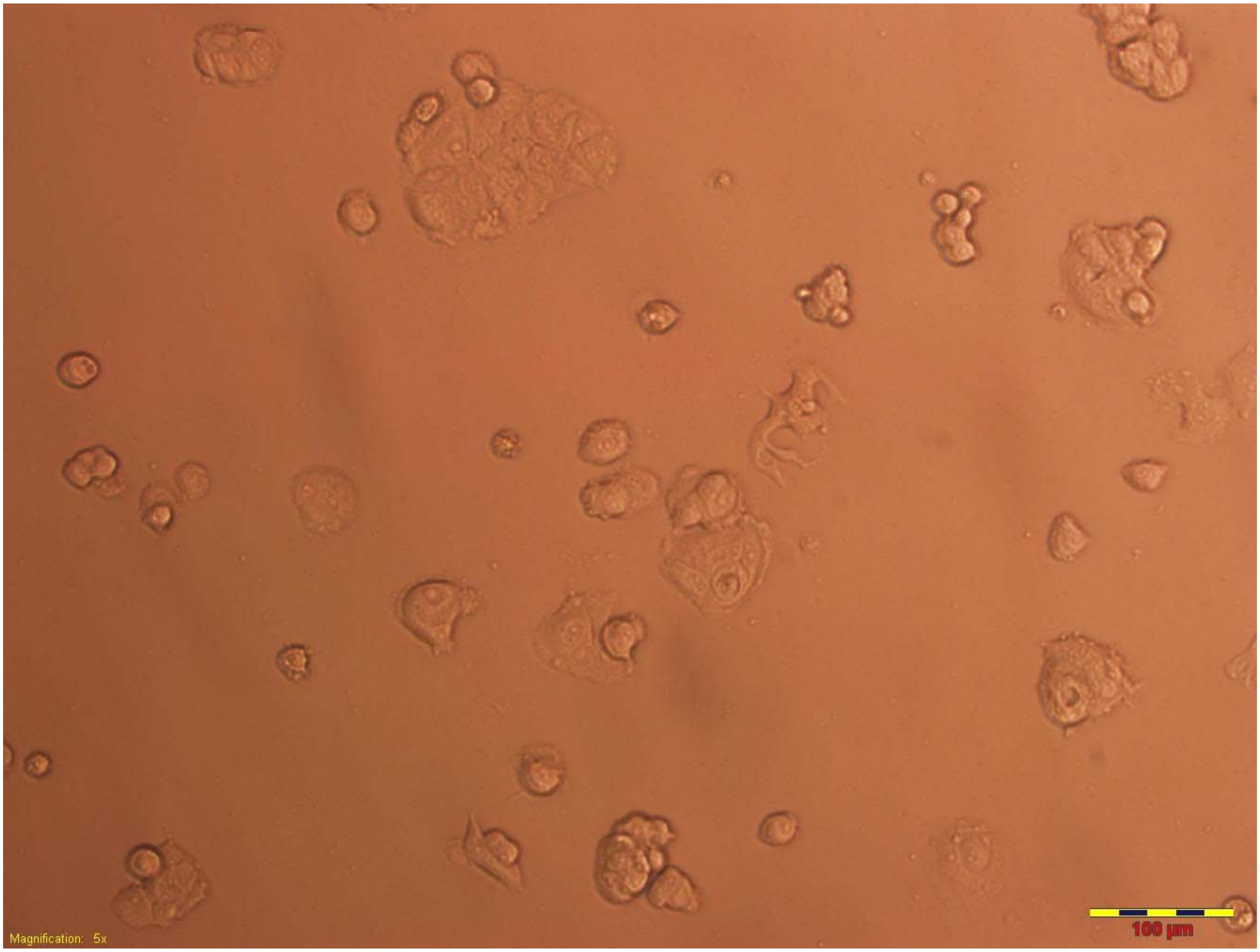

Figure S21. Bioprinted MCF7 breast cancer cells 72 hours post bioprinting, not stained

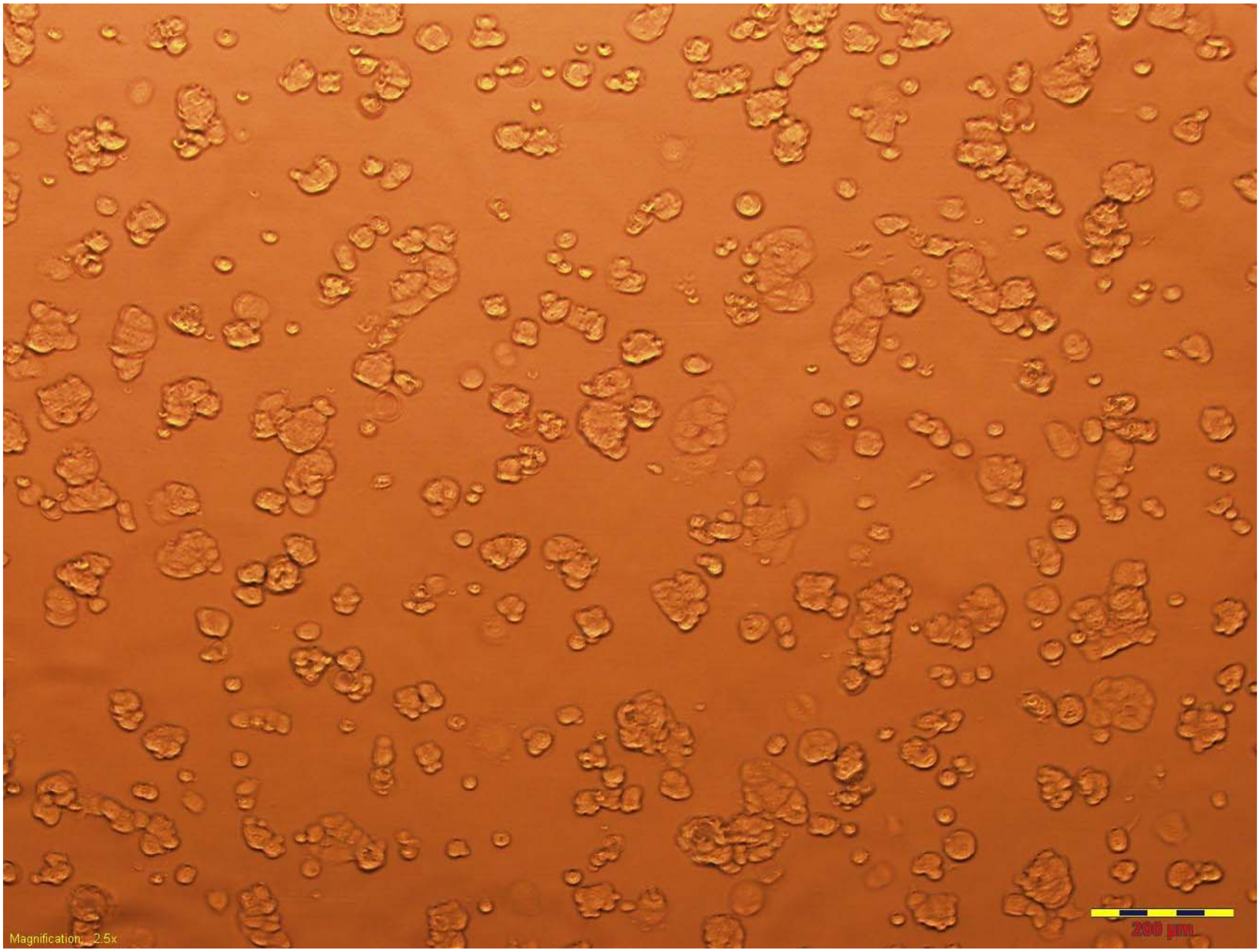

Figure S22. Manually seeded MCF7 breast cancer cells, prior to bioprinting, not stained

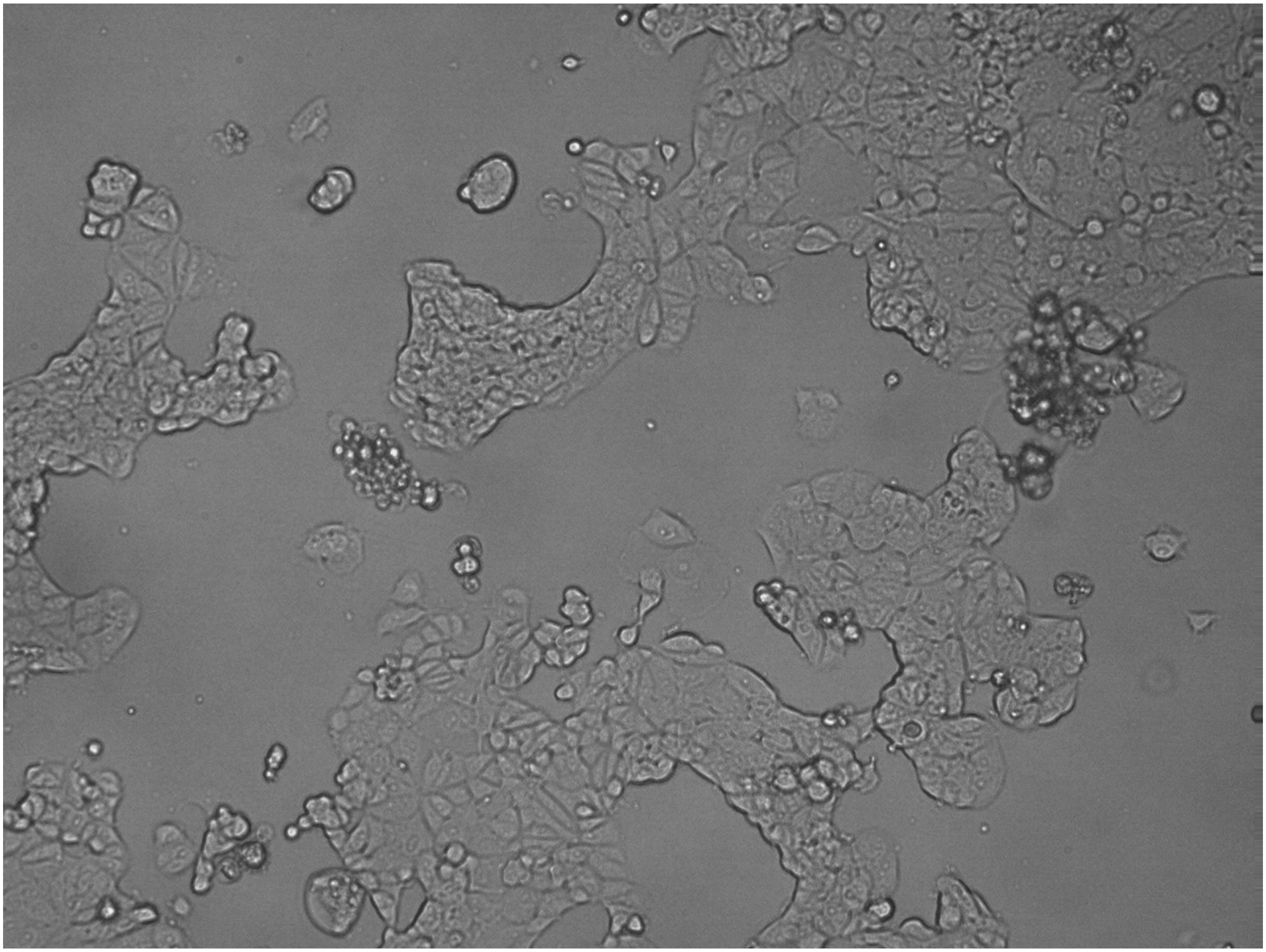

Figure S23. Bioprinted MCF7 breast cancer cells 7 days post bioprinting, not stained (0.25X)

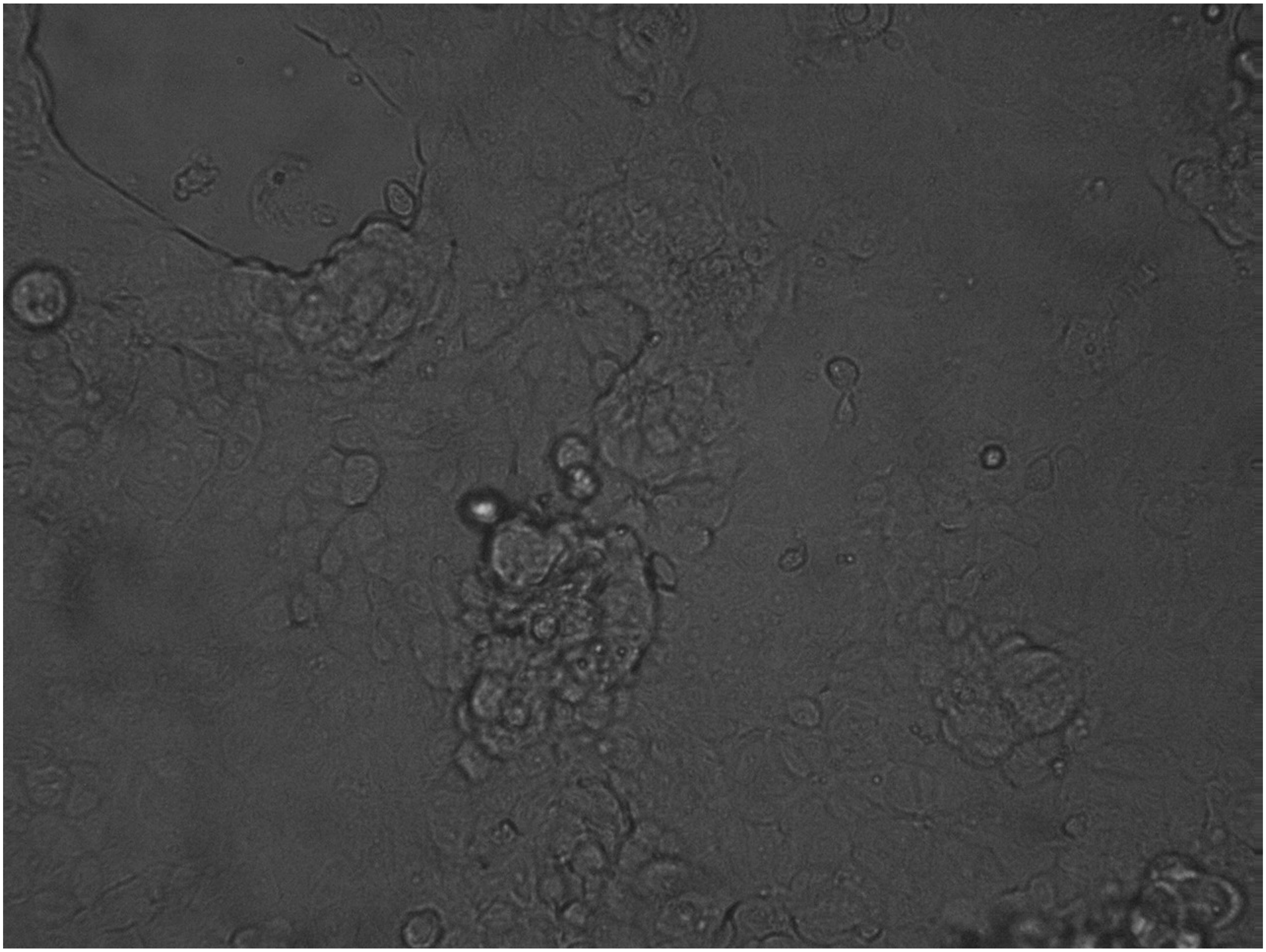

Figure S24. Manually seeded MCF7 breast cancer cells days post bioprinting, not stained (0.25x)

A

B

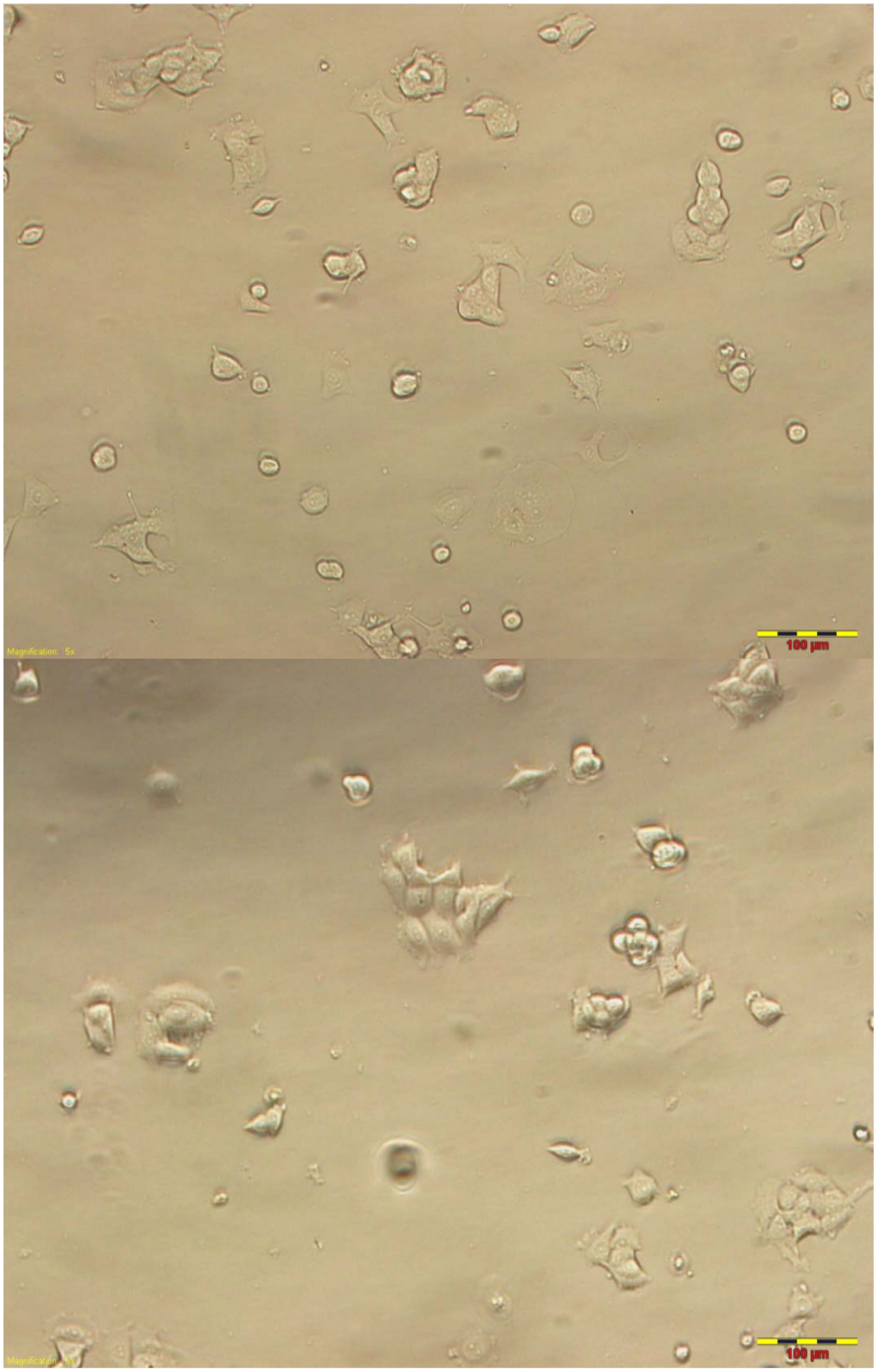

Figure S25. (A) Bioprinted and (B) Manually seeded MCF7 breast cancer cells.
