# Supplementary material for: Thermal Bioprinting Causes Ample Alterations of Expression of LUCAT1, IL6, CCL26, and NRN1L Genes and Massive Phosphorylation of Critical Oncogenic Drug Resistance Pathways in Breast Cancer Cells
Source: Front Bioeng Biotechnol. 2020 Feb 21;8:82. doi: 10.3389/fbioe.2020.00082 (PMC7047130; doi:10.3389/fbioe.2020.00082)
Supplement: Supplementary file 2 [file Table_1.pdf]

“Thermal Bioprinting Causes Ample Alterations of Expression of LUCAT1, IL6, CCL26 and NRN1L Genes and Massive Phosphorylation of Critical Oncogenic Drug Resistance Pathways in Breast Cancer Cells”

Supplemental Information - Part II

List of Tables

Table 1. Differentially expressed genes of bioprinted MCF7 breast cancer cells (Upregulated)……………………………………………………………………………1-7

Table 2. Differentially expressed genes of bioprinted MCF7 breast cancer cells (Downregulated)……………………………………………………………………...….8-13

Table 1. Differentially expressed genes of bioprinted MCF7 breast cancer cells (Upregulated)

Gene Name	Chromosome	Sample1	Sample2	Status	p-value	q-value
FBLI+A2:B208M1	chr1	MS	BP	OK	0.00025	0.00691968
GRHL3	chr1	MS	BP	OK	0.0018	0.0260298
SERINC2	chr1	MS	BP	OK	0.0005	0.0110415
MAP7D1	chr1	MS	BP	OK	0.00045	0.0102711
ARTN	chr1	MS	BP	OK	0.0034	0.0395772
PLEKHO1	chr1	MS	BP	OK	0.00195	0.0270787
SPRR1B	chr1	MS	BP	OK	0.00085	0.0157083
IL6R	chr1	MS	BP	OK	0.0009	0.0162159
TGFB2	chr1	MS	BP	OK	0.00025	0.00691968
SCCPDH	chr1	MS	BP	OK	5e-05	0.00199251
DHRS3	chr1	MS	BP	OK	0.00065	0.013144
NBPF1	chr1	MS	BP	OK	5e-05	0.00199251
IFFO2	chr1	MS	BP	OK	5e-05	0.00199251

RAP1GAP	chr1	MS	BP	OK	5e-05	0.00199251
MOB3C	chr1	MS	BP	OK	0.00035	0.00873857
LRP8	chr1	MS	BP	OK	5e-05	0.00199251
GBP2	chr1	MS	BP	OK	0.0008	0.015011
BCAR3	chr1	MS	BP	OK	5e-05	0.00199251
GCLM	chr1	MS	BP	OK	5e-05	0.00199251
PHTF1	chr1	MS	BP	OK	5e-05	0.00199251
S100A7	chr1	MS	BP	OK	0.00225	0.0301165
S100A6	chr1	MS	BP	OK	5e-05	0.00199251
SELL	chr1	MS	BP	OK	0.00215	0.0290001
C1orf116	chr1	MS	BP	OK	0.00085	0.0157083
DUSP10	chr1	MS	BP	OK	0.00175	0.0256401
CAPN8	chr1	MS	BP	OK	0.0004	0.00950214
SIPA1L2	chr1	MS	BP	OK	5e-05	0.00199251
AKR1C1	chr10	MS	BP	OK	0.00025	0.00691968
AKR1C3	chr10	MS	BP	OK	5e-05	0.00199251
ZNF365	chr10	MS	BP	OK	0.0019	0.0266873
DDX21	chr10	MS	BP	OK	5e-05	0.00199251
PLAU	chr10	MS	BP	OK	0.0036	0.0406295
PAPSS2	chr10	MS	BP	OK	5e-05	0.00199251
HHEX	chr10	MS	BP	OK	0.00115	0.0191677
AKR1C2	chr10	MS	BP	OK	5e-05	0.00199251
NRP1	chr10	MS	BP	OK	5e-05	0.00199251
LINC01468	chr10	MS	BP	OK	0.00015	0.00477891
AIFM2	chr10	MS	BP	OK	0.0001	0.00345593
IDE	chr10	MS	BP	OK	5e-05	0.00199251
CD44	chr11	MS	BP	OK	5e-05	0.00199251
FJX1	chr11	MS	BP	OK	0.00205	0.0279907
STX3	chr11	MS	BP	OK	5e-05	0.00199251
PPP2R5B	chr11	MS	BP	OK	0.0005	0.0110415
EHBP1L1	chr11	MS	BP	OK	5e-05	0.00199251
KLC2	chr11	MS	BP	OK	5e-05	0.00199251
GAL	chr11	MS	BP	OK	0.0006	0.0124414
MYEOV	chr11	MS	BP	OK	0.0032	0.037752
P2RY6	chr11	MS	BP	OK	5e-05	0.00199251
TMEM45B	chr11	MS	BP	OK	5e-05	0.00199251
DUSP8	chr11	MS	BP	OK	0.00025	0.00691968
FOSL1	chr11	MS	BP	OK	0.00025	0.00691968
PGM2L1	chr11	MS	BP	OK	0.0002	0.00595328
ARRB1	chr11	MS	BP	OK	5e-05	0.00199251
GDPD5	chr11	MS	BP	OK	0.0003	0.0078591
CHORDC1	chr11	MS	BP	OK	5e-05	0.00199251

GPRC5A	chr12	MS	BP	OK	5e-05	0.00199251
EMP1	chr12	MS	BP	OK	0.00025	0.00691968
TXNRD1	chr12	MS	BP	OK	5e-05	0.00199251
TRAFD1	chr12	MS	BP	OK	5e-05	0.00199251
VDR	chr12	MS	BP	OK	0.0001	0.00345593
KRT80	chr12	MS	BP	OK	5e-05	0.00199251
KRT4	chr12	MS	BP	OK	5e-05	0.00199251
B4GALNT1	chr12	MS	BP	OK	5e-05	0.00199251
PHLDA1	chr12	MS	BP	OK	5e-05	0.00199251
NTN4	chr12	MS	BP	OK	5e-05	0.00199251
SLC41A2	chr12	MS	BP	OK	5e-05	0.00199251
NAA25	chr12	MS	BP	OK	5e-05	0.00199251
CLIP1	chr12	MS	BP	OK	5e-05	0.00199251
PITPNM2	chr12	MS	BP	OK	5e-05	0.00199251
RGCC	chr13	MS	BP	OK	0.00395	0.0432626
LMO7	chr13	MS	BP	OK	0.00015	0.00477891
HS6ST3	chr13	MS	BP	OK	5e-05	0.00199251
HSPH1	chr13	MS	BP	OK	5e-05	0.00199251
LIG4	chr13	MS	BP	OK	0.00015	0.00477891
ABHD4	chr14	MS	BP	OK	5e-05	0.00199251
SAMD4A	chr14	MS	BP	OK	0.0002	0.00595328
TTC9	chr14	MS	BP	OK	0.00015	0.00477891
BATF	chr14	MS	BP	OK	5e-05	0.00199251
EIF5	chr14	MS	BP	OK	5e-05	0.00199251
CEP170B	chr14	MS	BP	OK	5e-05	0.00199251
FERMT2	chr14	MS	BP	OK	5e-05	0.00199251
MAP3K9	chr14	MS	BP	OK	5e-05	0.00199251
PTPN21	chr14	MS	BP	OK	0.0003	0.0078591
C15orf48	chr15	MS	BP	OK	5e-05	0.00199251
TMOD2	chr15	MS	BP	OK	0.00025	0.00691968
FURIN	chr15	MS	BP	OK	5e-05	0.00199251
SYNM	chr15	MS	BP	OK	5e-05	0.00199251
ALDH1A3	chr15	MS	BP	OK	5e-05	0.00199251
MYO1E	chr15	MS	BP	OK	5e-05	0.00199251
STRA6	chr15	MS	BP	OK	5e-05	0.00199251
SEMA7A	chr15	MS	BP	OK	0.0007	0.0138573
CYP1A1	chr15	MS	BP	OK	5e-05	0.00199251
TNFRSF12A	chr16	MS	BP	OK	5e-05	0.00199251
PDP2	chr16	MS	BP	OK	0.00205	0.0279907
NRN1L	chr16	MS	BP	OK	5e-05	0.00199251
GAN	chr16	MS	BP	OK	5e-05	0.00199251
OSGIN1	chr16	MS	BP	OK	5e-05	0.00199251

CRISPLD2	chr16	MS	BP	OK	5e-05	0.00199251
KIAA0513	chr16	MS	BP	OK	5e-05	0.00199251
ZNF469	chr16	MS	BP	OK	5e-05	0.00199251
UNKL	chr16	MS	BP	OK	5e-05	0.00199251
NQO1	chr16	MS	BP	OK	5e-05	0.00199251
WSCD1	chr17	MS	BP	OK	0.0003	0.0078591
ADORA2B	chr17	MS	BP	OK	0.003	0.0363386
TRIM16L	chr17	MS	BP	OK	5e-05	0.00199251
ABCC3	chr17	MS	BP	OK	5e-05	0.00199251
PRKCA	chr17	MS	BP	OK	5e-05	0.00199251
SPHK1	chr17	MS	BP	OK	5e-05	0.00199251
MIR22,MIR22HG	chr17	MS	BP	OK	5e-05	0.00199251
ZNF594	chr17	MS	BP	OK	0.00115	0.0191677
ALOXE3	chr17	MS	BP	OK	0.00475	0.0493306
TRIM16	chr17	MS	BP	OK	5e-05	0.00199251
FLII	chr17	MS	BP	OK	5e-05	0.00199251
ALDH3A1	chr17	MS	BP	OK	0.00045	0.0102711
KRT13	chr17	MS	BP	OK	5e-05	0.00199251
KRT17	chr17	MS	BP	OK	5e-05	0.00199251
MPP3	chr17	MS	BP	OK	0.00415	0.0450098
MAP3K14	chr17	MS	BP	OK	0.00075	0.0144724
MAFG	chr17	MS	BP	OK	5e-05	0.00199251
RBBP8	chr18	MS	BP	OK	5e-05	0.00199251
LAMA3	chr18	MS	BP	OK	5e-05	0.00199251
DTNA	chr18	MS	BP	OK	0.0003	0.0078591
MAPK4	chr18	MS	BP	OK	0.0002	0.00595328
FSTL3	chr19	MS	BP	OK	0.00035	0.00873857
CNN2	chr19	MS	BP	OK	5e-05	0.00199251
TRIP10	chr19	MS	BP	OK	0.0002	0.00595328
ICAM1	chr19	MS	BP	OK	0.0024	0.0315039
KLF2	chr19	MS	BP	OK	0.0014	0.0218186
GDF15	chr19	MS	BP	OK	5e-05	0.00199251
PLEKHF1	chr19	MS	BP	OK	0.0027	0.0337403
CEACAM5	chr19	MS	BP	OK	5e-05	0.00199251
CEACAM6	chr19	MS	BP	OK	5e-05	0.00199251
BCL3	chr19	MS	BP	OK	5e-05	0.00199251
IGFL1	chr19	MS	BP	OK	0.004	0.0436539
ZNF823	chr19	MS	BP	OK	0.0017	0.0251181
PSG9	chr19	MS	BP	OK	0.0042	0.0453111
KCNN4	chr19	MS	BP	OK	5e-05	0.00199251
KLK6	chr19	MS	BP	OK	0.00095	0.0166987
CLIP4	chr2	MS	BP	OK	0.0015	0.023054

EPAS1	chr2	MS	BP	OK	5e-05	0.00199251
ACTG2	chr2	MS	BP	OK	5e-05	0.00199251
ATOH8	chr2	MS	BP	OK	0.0005	0.0110415
SLC9A2	chr2	MS	BP	OK	0.00015	0.00477891
INHBB	chr2	MS	BP	OK	5e-05	0.00199251
FMNL2	chr2	MS	BP	OK	0.00025	0.00691968
ITGAV	chr2	MS	BP	OK	5e-05	0.00199251
MYO1B	chr2	MS	BP	OK	5e-05	0.00199251
NABP1	chr2	MS	BP	OK	5e-05	0.00199251
NRP2	chr2	MS	BP	OK	0.0016	0.0241361
CCNYL1	chr2	MS	BP	OK	5e-05	0.00199251
PSMD1	chr2	MS	BP	OK	5e-05	0.00199251
UGT1A1,UGT1A10,UGT1A3,UGT1A4,UGT1A5,UGT1A6,UGT1A7,UGT1A8,UGT1A9	chr2	MS	BP	OK	5e-05	0.00199251
CAPN13	chr2	MS	BP	OK	0.0008	0.015011
FAM98A	chr2	MS	BP	OK	5e-05	0.00199251
CYP1B1	chr2	MS	BP	OK	5e-05	0.00199251
PSME4	chr2	MS	BP	OK	5e-05	0.00199251
ZNF514	chr2	MS	BP	OK	0.00075	0.0144724
FHL2	chr2	MS	BP	OK	5e-05	0.00199251
MALL	chr2	MS	BP	OK	5e-05	0.00199251
TFPI	chr2	MS	BP	OK	5e-05	0.00199251
HECW2	chr2	MS	BP	OK	5e-05	0.00199251
ABCB6	chr2	MS	BP	OK	5e-05	0.00199251
AP1S3	chr2	MS	BP	OK	0.0002	0.00595328
BPIFB1	chr20	MS	BP	OK	0.00355	0.0404007
SRXN1	chr20	MS	BP	OK	5e-05	0.00199251
LZTS3	chr20	MS	BP	OK	0.00435	0.0463167
SLC4A11	chr20	MS	BP	OK	0.0008	0.015011
THBD	chr20	MS	BP	OK	5e-05	0.00199251
TMPRSS2	chr21	MS	BP	OK	5e-05	0.00199251
SEC14L2	chr22	MS	BP	OK	0.0046	0.0483474
HMOX1	chr22	MS	BP	OK	5e-05	0.00199251
PANX2	chr22	MS	BP	OK	5e-05	0.00199251
MAPK8IP2	chr22	MS	BP	OK	0.00295	0.0359465
LIF	chr22	MS	BP	OK	0.0004	0.00950214
TRNT1	chr3	MS	BP	OK	5e-05	0.00199251
LMCD1	chr3	MS	BP	OK	5e-05	0.00199251
PPARG	chr3	MS	BP	OK	5e-05	0.00199251
SLC6A6	chr3	MS	BP	OK	5e-05	0.00199251

ATP1B3	chr3	MS	BP	OK	5e-05	0.00199251
MFSD1	chr3	MS	BP	OK	5e-05	0.00199251
IQCJ,IQCJ-SCHIP1,SCHIP1	chr3	MS	BP	OK	0.0032	0.037752
KCCAT211	chr3	MS	BP	OK	0.00215	0.0290001
ST6GAL1	chr3	MS	BP	OK	0.00125	0.0201217
PRRT3	chr3	MS	BP	OK	5e-05	0.00199251
SLC4A7	chr3	MS	BP	OK	5e-05	0.00199251
MGLL	chr3	MS	BP	OK	5e-05	0.00199251
LXN	chr3	MS	BP	OK	5e-05	0.00199251
TNFSF10	chr3	MS	BP	OK	0.0009	0.0162159
LIPH	chr3	MS	BP	OK	0.00385	0.0427025
CLDN1	chr3	MS	BP	OK	0.0005	0.0110415
LRRC15	chr3	MS	BP	OK	0.0011	0.0187429
PCYT1A	chr3	MS	BP	OK	5e-05	0.00199251
NAT8L	chr4	MS	BP	OK	5e-05	0.00199251
ANXA3	chr4	MS	BP	OK	5e-05	0.00199251
AGPAT9	chr4	MS	BP	OK	5e-05	0.00199251
TLR2	chr4	MS	BP	OK	0.0014	0.0218186
PALLD	chr4	MS	BP	OK	5e-05	0.00199251
SNX25	chr4	MS	BP	OK	0.0014	0.0218186
ABCG2	chr4	MS	BP	OK	5e-05	0.00199251
GPRIN3	chr4	MS	BP	OK	0.0007	0.0138573
SLC7A11	chr4	MS	BP	OK	5e-05	0.00199251
UBE2QL1	chr5	MS	BP	OK	0.00185	0.0263173
FAM105A	chr5	MS	BP	OK	5e-05	0.00199251
RAI14	chr5	MS	BP	OK	5e-05	0.00199251
MAP1B	chr5	MS	BP	OK	5e-05	0.00199251
F2RL1	chr5	MS	BP	OK	0.0001	0.00345593
SLC12A2	chr5	MS	BP	OK	5e-05	0.00199251
TGFBI	chr5	MS	BP	OK	5e-05	0.00199251
SQSTM1	chr5	MS	BP	OK	5e-05	0.00199251
PLCXD3	chr5	MS	BP	OK	0.00035	0.00873857
FAM169A	chr5	MS	BP	OK	0.00305	0.0367623
LHFPL2	chr5	MS	BP	OK	5e-05	0.00199251
LUCAT1	chr5	MS	BP	OK	5e-05	0.00199251
RUNX2	chr6	MS	BP	OK	0.0004	0.00950214
RAB32	chr6	MS	BP	OK	0.00035	0.00873857
TUBB2A	chr6	MS	BP	OK	0.0021	0.028546
CCND3	chr6	MS	BP	OK	5e-05	0.00199251
TNFRSF21	chr6	MS	BP	OK	5e-05	0.00199251
SLC17A5	chr6	MS	BP	OK	0.0014	0.0218186

ME1	chr6	MS	BP	OK	5e-05	0.00199251
MAN1A1	chr6	MS	BP	OK	0.0002	0.00595328
FAM20C	chr7	MS	BP	OK	0.0002	0.00595328
IL6	chr7	MS	BP	OK	5e-05	0.00199251
UPP1	chr7	MS	BP	OK	5e-05	0.00199251
EGFR	chr7	MS	BP	OK	0.002	0.0274921
CROT	chr7	MS	BP	OK	0.00115	0.0191677
ORAI2	chr7	MS	BP	OK	0.00015	0.00477891
IFRD1	chr7	MS	BP	OK	5e-05	0.00199251
AKR1B10	chr7	MS	BP	OK	5e-05	0.00199251
MTRNR2L6	chr7	MS	BP	OK	0.0016	0.0241361
ZYX	chr7	MS	BP	OK	5e-05	0.00199251
STX1A	chr7	MS	BP	OK	0.0002	0.00595328
CCL26	chr7	MS	BP	OK	5e-05	0.00199251
KIAA1549	chr7	MS	BP	OK	5e-05	0.00199251
KIAA1147	chr7	MS	BP	OK	5e-05	0.00199251
CLN8	chr8	MS	BP	OK	5e-05	0.00199251
SDCBP	chr8	MS	BP	OK	5e-05	0.00199251
RDH10	chr8	MS	BP	OK	5e-05	0.00199251
PSCA	chr8	MS	BP	OK	0.0004	0.00950214
CSGALNACT1	chr8	MS	BP	OK	0.0006	0.0124414
TNFRSF10A	chr8	MS	BP	OK	0.00295	0.0359465
LOXL2	chr8	MS	BP	OK	5e-05	0.00199251
ANKRD46	chr8	MS	BP	OK	0.00035	0.00873857
TNFRSF11B	chr8	MS	BP	OK	5e-05	0.00199251
ST3GAL1	chr8	MS	BP	OK	5e-05	0.00199251
JRK	chr8	MS	BP	OK	0.0003	0.0078591
SCRIB	chr8	MS	BP	OK	5e-05	0.00199251
CPSF1	chr8	MS	BP	OK	5e-05	0.00199251
DNAJA1	chr9	MS	BP	OK	5e-05	0.00199251
C9orf47,S1PR3	chr9	MS	BP	OK	5e-05	0.00199251
WNK2	chr9	MS	BP	OK	5e-05	0.00199251
C9orf3	chr9	MS	BP	OK	5e-05	0.00199251
CYSRT1	chr9	MS	BP	OK	0.0012	0.0195483
AQP3	chr9	MS	BP	OK	5e-05	0.00199251
NOL6	chr9	MS	BP	OK	5e-05	0.00199251
FAM219A	chr9	MS	BP	OK	5e-05	0.00199251
AAED1	chr9	MS	BP	OK	0.002	0.0274921
PTGR1	chr9	MS	BP	OK	5e-05	0.00199251
NHS	chrX	MS	BP	OK	0.00025	0.00691968
FAM155B	chrX	MS	BP	OK	0.004	0.0436539
RAI2	chrX	MS	BP	OK	0.0025	0.0321271

LOC401585	chrX	MS	BP	OK	0.00155	0.0235561
RGAG4	chrX	MS	BP	OK	5e-05	0.00199251
L1CAM	chrX	MS	BP	OK	5e-05	0.00199251
G6PD	chrX	MS	BP	OK	5e-05	0.00199251

Table 2. Differentially expressed genes of bioprinted MCF7 breast cancer cells (Downregulated)

Gene Name	Chromosome	Sample1	Sample2	Status	Value1	Value2
GABRD	chr1	MS	BP	OK	0.00245	0.0317514
MST1P2	chr1	MS	BP	OK	0.00185	0.0263173
CYP4B1	chr1	MS	BP	OK	0.00055	0.0117841
CDKN2C	chr1	MS	BP	OK	0.0003	0.0078591
CYR61	chr1	MS	BP	OK	5e-05	0.00199251
PHGDH	chr1	MS	BP	OK	5e-05	0.00199251
HIST2H2AC	chr1	MS	BP	OK	0.00035	0.00873857
NOS1AP	chr1	MS	BP	OK	0.00105	0.0180164
IER5	chr1	MS	BP	OK	5e-05	0.00199251
NR5A2	chr1	MS	BP	OK	5e-05	0.00199251
PCAT6	chr1	MS	BP	OK	0.0048	0.0496812
PPFIA4	chr1	MS	BP	OK	5e-05	0.00199251
LINC00467	chr1	MS	BP	OK	0.00475	0.0493306
ATF3	chr1	MS	BP	OK	0.0003	0.0078591
SPATA17	chr1	MS	BP	OK	0.0032	0.037752
MEGF6	chr1	MS	BP	OK	0.0001	0.00345593
MFAP2	chr1	MS	BP	OK	0.0044	0.0466461
TCEA3	chr1	MS	BP	OK	0.00425	0.0456493
STMN1	chr1	MS	BP	OK	5e-05	0.00199251
FAM46B	chr1	MS	BP	OK	0.0001	0.00345593
GRIK3	chr1	MS	BP	OK	0.0036	0.0406295
JUN	chr1	MS	BP	OK	5e-05	0.00199251
ITGB3BP	chr1	MS	BP	OK	0.0008	0.015011
RORC	chr1	MS	BP	OK	5e-05	0.00199251
TMEM254	chr10	MS	BP	OK	0.0004	0.00950214
ADRA2A	chr10	MS	BP	OK	0.00185	0.0263173
DPYSL4	chr10	MS	BP	OK	5e-05	0.00199251
ST8SIA6	chr10	MS	BP	OK	0.0021	0.028546
EGR2	chr10	MS	BP	OK	0.003	0.0363386
AFAP1L2	chr10	MS	BP	OK	0.00015	0.00477891

GFRA1	chr10	MS	BP	OK	5e-05	0.00199251
BNIP3	chr10	MS	BP	OK	5e-05	0.00199251
IFITM1	chr11	MS	BP	OK	0.0001	0.00345593
ZBED5-AS1	chr11	MS	BP	OK	0.0026	0.0328939
MIR210HG	chr11	MS	BP	OK	0.0026	0.0328939
ASCL2	chr11	MS	BP	OK	0.0008	0.015011
LRP4	chr11	MS	BP	OK	5e-05	0.00199251
PGR	chr11	MS	BP	OK	5e-05	0.00199251
FXYD2,FXYD6,FXYD6-FXYD2	chr11	MS	BP	OK	0.001	0.0174274
TMEM218	chr11	MS	BP	OK	0.0001	0.00345593
METTL7A	chr12	MS	BP	OK	0.0002	0.00595328
NR4A1	chr12	MS	BP	OK	5e-05	0.00199251
LRP1	chr12	MS	BP	OK	0.0002	0.00595328
ASCL1	chr12	MS	BP	OK	5e-05	0.00199251
P2RX2	chr12	MS	BP	OK	0.00485	0.0499873
ING4	chr12	MS	BP	OK	0.0004	0.00950214
CIT	chr12	MS	BP	OK	0.00155	0.0235561
SHISA2	chr13	MS	BP	OK	0.00045	0.0102711
SMAD9	chr13	MS	BP	OK	0.00245	0.0317514
ADPRHL1	chr13	MS	BP	OK	0.0005	0.0110415
DHRS2	chr14	MS	BP	OK	5e-05	0.00199251
PCK2	chr14	MS	BP	OK	5e-05	0.00199251
FOS	chr14	MS	BP	OK	5e-05	0.00199251
EVL	chr14	MS	BP	OK	5e-05	0.00199251
LINC00641	chr14	MS	BP	OK	0.00045	0.0102711
C14orf93	chr14	MS	BP	OK	0.0033	0.0386707
SLC7A8	chr14	MS	BP	OK	0.0003	0.0078591
ALDH6A1	chr14	MS	BP	OK	5e-05	0.00199251
EFCAB11	chr14	MS	BP	OK	0.00045	0.0102711
DEGS2	chr14	MS	BP	OK	5e-05	0.00199251
LOC100288637	chr15	MS	BP	OK	0.0003	0.0078591
GCHFR	chr15	MS	BP	OK	0.00225	0.0301165
RCCD1	chr15	MS	BP	OK	5e-05	0.00199251
RASGRP1	chr15	MS	BP	OK	0.00105	0.0180164
PIF1	chr15	MS	BP	OK	5e-05	0.00199251
DET1	chr15	MS	BP	OK	0.0019	0.0266873
MT2A	chr16	MS	BP	OK	5e-05	0.00199251
MT1X	chr16	MS	BP	OK	5e-05	0.00199251
LINC01569	chr16	MS	BP	OK	0.002	0.0274921
LDHD	chr16	MS	BP	OK	0.00015	0.00477891
SLC22A31	chr16	MS	BP	OK	0.00275	0.0341906

FAM64A	chr17	MS	BP	OK	5e-05	0.00199251
RAPGEFL1	chr17	MS	BP	OK	5e-05	0.00199251
FZD2	chr17	MS	BP	OK	0.00045	0.0102711
MAP2K6	chr17	MS	BP	OK	0.00195	0.0270787
YBX2	chr17	MS	BP	OK	5e-05	0.00199251
TP53	chr17	MS	BP	OK	5e-05	0.00199251
TMEM107	chr17	MS	BP	OK	5e-05	0.00199251
HS3ST3A1	chr17	MS	BP	OK	5e-05	0.00199251
LYRM9	chr17	MS	BP	OK	5e-05	0.00199251
ALDOC	chr17	MS	BP	OK	5e-05	0.00199251
PROCA1	chr17	MS	BP	OK	0.00455	0.047863
PRR15L	chr17	MS	BP	OK	5e-05	0.00199251
TEX14	chr17	MS	BP	OK	5e-05	0.00199251
AMZ2P1	chr17	MS	BP	OK	0.0006	0.0124414
SDK2	chr17	MS	BP	OK	0.0006	0.0124414
GADD45B	chr19	MS	BP	OK	5e-05	0.00199251
TNFAIP8L1	chr19	MS	BP	OK	0.0014	0.0218186
IER2	chr19	MS	BP	OK	5e-05	0.00199251
CASP14	chr19	MS	BP	OK	0.0004	0.00950214
ARHGAP33	chr19	MS	BP	OK	5e-05	0.00199251
BCKDHA	chr19	MS	BP	OK	5e-05	0.00199251
FOSB	chr19	MS	BP	OK	5e-05	0.00199251
CCDC61	chr19	MS	BP	OK	0.0018	0.0260298
PPP1R15A	chr19	MS	BP	OK	5e-05	0.00199251
GAMT	chr19	MS	BP	OK	0.0018	0.0260298
PLIN5	chr19	MS	BP	OK	0.0005	0.0110415
EPOR	chr19	MS	BP	OK	0.00465	0.0487056
ZNF850	chr19	MS	BP	OK	0.0043	0.0459443
ZNF541	chr19	MS	BP	OK	0.00095	0.0166987
TMEM143	chr19	MS	BP	OK	0.0006	0.0124414
DBP	chr19	MS	BP	OK	5e-05	0.00199251
KLK11	chr19	MS	BP	OK	5e-05	0.00199251
ZSCAN18	chr19	MS	BP	OK	0.00015	0.00477891
KLF11	chr2	MS	BP	OK	5e-05	0.00199251
GREB1	chr2	MS	BP	OK	5e-05	0.00199251
THNSL2	chr2	MS	BP	OK	0.0009	0.0162159
SLC4A10	chr2	MS	BP	OK	0.0006	0.0124414
PDK1	chr2	MS	BP	OK	5e-05	0.00199251
FAM117B	chr2	MS	BP	OK	0.0019	0.0266873
KCNE4	chr2	MS	BP	OK	5e-05	0.00199251
SLC8A1	chr2	MS	BP	OK	0.00425	0.0456493
LINC00342	chr2	MS	BP	OK	0.0027	0.0337403

CXCR4	chr2	MS	BP	OK	5e-05	0.00199251
RBM43	chr2	MS	BP	OK	0.0017	0.0251181
NR4A2	chr2	MS	BP	OK	5e-05	0.00199251
ABCA12	chr2	MS	BP	OK	5e-05	0.00199251
SPAG4	chr20	MS	BP	OK	0.00035	0.00873857
FAM83D	chr20	MS	BP	OK	5e-05	0.00199251
LINC00494	chr20	MS	BP	OK	5e-05	0.00199251
MAFB	chr20	MS	BP	OK	5e-05	0.00199251
LINC01522	chr20	MS	BP	OK	5e-05	0.00199251
SYCP2	chr20	MS	BP	OK	5e-05	0.00199251
DSCR8	chr21	MS	BP	OK	0.0035	0.0400552
PCP4	chr21	MS	BP	OK	5e-05	0.00199251
TMPRSS3	chr21	MS	BP	OK	0.0001	0.00345593
SIK1	chr21	MS	BP	OK	5e-05	0.00199251
C21orf58	chr21	MS	BP	OK	0.0001	0.00345593
ADM2	chr22	MS	BP	OK	0.00345	0.0397807
PLA2G6	chr22	MS	BP	OK	0.00265	0.033354
DNAL4	chr22	MS	BP	OK	0.00235	0.0310139
TTLL1	chr22	MS	BP	OK	0.00135	0.0212014
DZIP3	chr3	MS	BP	OK	0.0025	0.0321271
HES1	chr3	MS	BP	OK	5e-05	0.00199251
SRGAP3	chr3	MS	BP	OK	0.0001	0.00345593
CSRNP1	chr3	MS	BP	OK	5e-05	0.00199251
MST1	chr3	MS	BP	OK	0.0042	0.0453111
CISH	chr3	MS	BP	OK	0.00055	0.0117841
KIAA1407	chr3	MS	BP	OK	0.0043	0.0459443
ALG1L	chr3	MS	BP	OK	0.00435	0.0463167
H1FX	chr3	MS	BP	OK	5e-05	0.00199251
FRAS1	chr4	MS	BP	OK	0.00015	0.00477891
ENPEP	chr4	MS	BP	OK	0.00455	0.047863
NMU	chr4	MS	BP	OK	0.0044	0.0466461
UGT2B15	chr4	MS	BP	OK	0.0001	0.00345593
HMGB2	chr4	MS	BP	OK	5e-05	0.00199251
FBXO4	chr5	MS	BP	OK	0.00135	0.0212014
GPX8	chr5	MS	BP	OK	0.002	0.0274921
CENPH	chr5	MS	BP	OK	0.0001	0.00345593
KIF20A	chr5	MS	BP	OK	0.0037	0.0415283
EGR1	chr5	MS	BP	OK	5e-05	0.00199251
PTTG1	chr5	MS	BP	OK	5e-05	0.00199251
HMMR	chr5	MS	BP	OK	5e-05	0.00199251
SEPP1	chr5	MS	BP	OK	0.00235	0.0310139
ARRDC3	chr5	MS	BP	OK	5e-05	0.00199251

NREP	chr5	MS	BP	OK	0.00305	0.0367623
FAXDC2	chr5	MS	BP	OK	0.00045	0.0102711
DUSP1	chr5	MS	BP	OK	5e-05	0.00199251
DEF6	chr6	MS	BP	OK	0.00035	0.00873857
PNRC1	chr6	MS	BP	OK	5e-05	0.00199251
KCNK5	chr6	MS	BP	OK	0.0009	0.0162159
CTGF	chr6	MS	BP	OK	5e-05	0.00199251
SGK1	chr6	MS	BP	OK	0.00035	0.00873857
CITED2	chr6	MS	BP	OK	5e-05	0.00199251
RAMP3	chr7	MS	BP	OK	0.00035	0.00873857
RADIL	chr7	MS	BP	OK	0.00045	0.0102711
LINC00174	chr7	MS	BP	OK	0.0008	0.015011
SEMA3A	chr7	MS	BP	OK	0.00045	0.0102711
ASNS	chr7	MS	BP	OK	5e-05	0.00199251
RASA4	chr7	MS	BP	OK	0.0006	0.0124414
PLXNA4	chr7	MS	BP	OK	0.0002	0.00595328
BNIP3L	chr8	MS	BP	OK	5e-05	0.00199251
EPHX2	chr8	MS	BP	OK	0.003	0.0363386
C8orf46	chr8	MS	BP	OK	0.0026	0.0328939
DEPTOR	chr8	MS	BP	OK	5e-05	0.00199251
TRIB1	chr8	MS	BP	OK	5e-05	0.00199251
MYC	chr8	MS	BP	OK	5e-05	0.00199251
EGR3	chr8	MS	BP	OK	5e-05	0.00199251
PLAT	chr8	MS	BP	OK	0.00025	0.00691968
GEM	chr8	MS	BP	OK	0.0008	0.015011
NDRG1	chr8	MS	BP	OK	5e-05	0.00199251
TSNARE1	chr8	MS	BP	OK	0.00285	0.0351127
LINC01604	chr8	MS	BP	OK	0.0012	0.0195483
PARP10	chr8	MS	BP	OK	0.0001	0.00345593
CA9	chr9	MS	BP	OK	5e-05	0.00199251
TMEM8B	chr9	MS	BP	OK	0.0042	0.0453111
GADD45G	chr9	MS	BP	OK	0.00025	0.00691968
NR4A3	chr9	MS	BP	OK	0.00105	0.0180164
ZNF618	chr9	MS	BP	OK	0.0007	0.0138573
COL27A1	chr9	MS	BP	OK	0.00015	0.00477891
CNTRL	chr9	MS	BP	OK	5e-05	0.00199251
OLFML2A	chr9	MS	BP	OK	0.0031	0.037109
ASS1	chr9	MS	BP	OK	5e-05	0.00199251
PTPRD	chr9	MS	BP	OK	0.0004	0.00950214
ANKRD18A	chr9	MS	BP	OK	0.00075	0.0144724
C5	chr9	MS	BP	OK	0.00045	0.0102711
GYG2	chrX	MS	BP	OK	0.00195	0.0270787

KLHL4	chrX	MS	BP	OK	0.0002	0.00595328
TMEM187	chrX	MS	BP	OK	5e-05	0.00199251
PNPLA4	chrX	MS	BP	OK	0.0021	0.028546
MID1	chrX	MS	BP	OK	0.0031	0.037109
ZMAT1	chrX	MS	BP	OK	0.0002	0.00595328
GPC3	chrX	MS	BP	OK	0.00235	0.0310139
SOX3	chrX	MS	BP	OK	5e-05	0.00199251
CH17-340M24.3	chrX	MS	BP	OK	0.0042	0.0453111
